# Reduction in SH-SY5Y Cell Stress Induced by Corticosterone and Attenuation of the Inflammatory Response in RAW 264.7 Cells Using Endomorphin Analogs

**DOI:** 10.3390/biomedicines13071774

**Published:** 2025-07-20

**Authors:** Renata Perlikowska, Angelika Długosz-Pokorska, Małgorzata Domowicz, Sylwia Grabowicz, Mariusz Stasiołek, Małgorzata Zakłos-Szyda

**Affiliations:** 1 Department of Biomolecular Chemistry, Faculty of Medicine, Medical University of Lodz, Mazowiecka 6/8, 92-215 Lodz, Poland; angelika.dlugosz@umed.lodz.pl (A.D.-P.); sylwiagrabowicz.farm@gmail.com (S.G.); 2 Department of Neurology, Faculty of Medicine, Medical University of Lodz, Kosciuszki Street 4, 90-419 Lodz, Poland; malgorzata.domowicz@umed.lodz.pl (M.D.); mariusz.stasiolek@umed.lodz.pl (M.S.); 3 Institute of Molecular and Industrial Biotechnology, Faculty of Biotechnology and Food Sciences, Lodz University of Technology, Stefanowskiego 2/22, 90-537 Lodz, Poland; malgorzata.zaklos-szyda@p.lodz.pl

**Keywords:** neuroprotection, opioid peptides, cell injury, neuronal damage, depression, cell lines, inflammation

## Abstract

**Background:** To identify drug candidates that reduce cellular stress, linear peptides known as endomorphin (EM) analogs containing proline surrogates in position 2 were tested in in vitro injury models induced by corticosterone (CORT). **Methods:** In this study, neuroblastoma (SH-SY5Y) cells were treated with CORT and synthesized peptides, and then the cell viability and morphology, reactive oxygen species production (ROS), mitochondrial membrane potential (ΔΨm), adenosine triphosphate (ATP), and intracellular calcium ion [Ca^2+^]_i_ levels were evaluated. We also conducted an in-depth analysis of the apoptosis markers using quantitative real-time PCR (qPCR). Finally, we explore the brain-derived neurotrophic factor (BDNF) expression (qPCR) and protein levels (ELI-SA and Western blot). **Results:** The strongest neuroprotective effect in the CORT-induced stress model was shown by peptide 3 and peptide 7 (in the following sequence Tyr-Inp-Trp-Phe-NH2 and Tyr-Inp-Phe-Phe-NH2, respectively). These peptides significantly improved cell viability and reduced oxidative stress in CORT-treated cells. **Conclusions:** Their neuroprotective potential appears linked to anti-apoptotic effects, along with in-creased BDNF expression. Moreover, in the lipopolysaccharide (LPS)- and interferon-γ (IFN-γ)-induced damage model in macrophage RAW 264.7 cells, these two peptides reduced the secretion of inflammatory mediators nitric oxide (NO), tumor necrosis factor-α (TNF-α), and interleukin-6 (IL-6). Peptides exhibiting both neuroprotective and anti-inflammatory properties warrant further investigation as potential therapeutic agents.

## 1. Introduction

The mechanisms responsible for neuronal damage induced by stress remained partially unclear, prompting ongoing research efforts to elucidate these mechanisms. Stress-induced impairment, particularly chronic or severe stress, has been associated with an increased risk of various diseases and health conditions, including cardiovascular problems, gastrointestinal issues, immune system dysfunction, endocrine disorders, and neurological disorders [1]. However, in the presented experiments and research, we focused on stress as a known risk factor for mental health conditions such as depression.

The hypothalamic–pituitary–adrenal (HPA) axis plays a central role in the pathophysiology of depression, a disorder traditionally managed with psychotherapy and pharmacological agents targeting monoaminergic systems [2,3,4]. Despite the availability of newer monoamine reuptake inhibitors and receptor antagonists, current treatments are still based on drugs discovered in the 1960s and early 1970s, and remain limited by adverse effects such as sedation, gastrointestinal disturbances, sexual dysfunction, or weight gain.

The HPA axis regulates the physiological response to stress and modulates key processes including metabolism, immune function, digestion, mood, and sexual behavior. Upon stress exposure, glucocorticoids (GCs)—primarily cortisol in humans and corticosterone (CORT) in rodents—are secreted by the adrenal cortex. These hormones influence neurogenesis, synaptic plasticity, and neuronal survival, and are essential for restoring homeostasis. However, chronic stress can lead to HPA axis hyperactivity and dysregulation, resulting in sustained elevations of circulating GCs. Prolonged GC exposure has been implicated in immune suppression [5], metabolic disturbances [6], osteoporosis [7], mood disorders [8], hippocampal atrophy [9], and cognitive impairment [10,11,12].

Since we know that excessive GC levels may have a huge impact on neuron condition and lead to their damage in several regions of the brain, we proposed to search for a drug candidate with neuroprotective potency, using an in vitro model of neuronal damage caused by CORT. Using CORT in cell models offers a controlled and consistent method to study how stress affects cellular processes.

In trying to develop new antidepressant medicines, opioid peptides will be used as hypothetical therapeutic agents. Opioid receptors are widely expressed in the central and peripheral nervous system and the non-neuronal tissues [13]. Ligands for these receptors are compounds of exogenous and endogenous origin, with different structures and functions; among them are opioid peptides (i.e., enkephalins, endorphins, and dynorphins). They remain essential for alleviating pain. However, in addition to their classic role in pain inhibition, opioid peptides are involved in physiological actions such as immune responses, feeding, drinking, locomotor activation, and the regulation of mood and memory [14]. Moreover, they show diverse biological effects, including cytoprotective and neuroprotective effects [15,16]. A growing body of evidence suggests that the opioid system, as well as opioid peptides, promote the survival of neurons during the development of the nervous system, influencing their proliferation and migration (see more in [16]). Moreover, our recent studies showed that two highly selective endogenous µ-opioid receptor (MOR) ligands, endomorphin-1 (EM-1, Tyr-Pro-Trp-Phe-NH_2_) and endomorphin-2 (EM-2, Tyr-Pro-Phe-Phe-NH_2_), significantly recovered the neurotoxicity induced by 6-hydroxydopamine (6-OHDA) in the human neuroblastoma cell line (SH-SY5Y) [17]. They increased cell viability (7–10%), and EM-1 (at the dose of 1 μM) showed a significant decrease in mitochondrial membrane potential (∆Ψm) depolarization, while EM-2 (at the dose of 1–10 μM) diminished caspase-3 activity.

**Figure 1:**
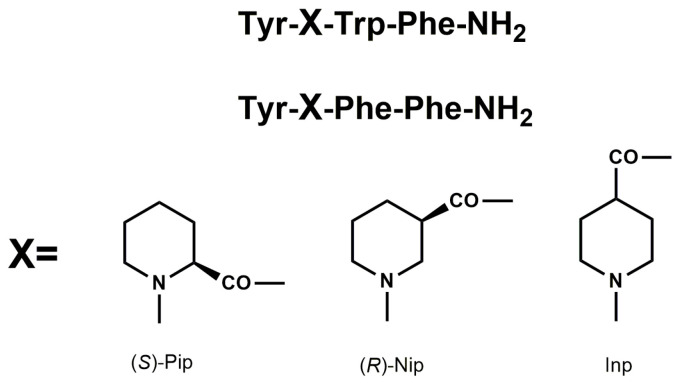
Structural modifications of parent peptides at position 2 with heterocyclic unnatural amino acids piperidine-2-, 3-, and 4-carboxylic acids ((*S*)-Pip, (*R*)-Nip, and Inp, respectively).

Endogenous and naturally occurring opioid peptides may serve as important leads for the design of peptide analogs with better pharmacodynamic and pharmacokinetic properties. Previously, we have described synthesis, together with biological evaluation of EM analogs, modified in position 2 by incorporation of unnatural amino acids with six-membered heterocyclic rings, such as piperidine-2-, 3-, and 4-carboxylic acids ((*S*)-Pip, (*R*)-Nip, and Inp, respectively) (Figure 1) [18,19,20].

Modified EM analogs showed enhanced μ-opioid receptor affinity, functional potency, metabolic stability, and in vivo antinociceptive effects after intracerebroventricular (icv) administration, which was completely reversed by β-funaltrexamine (β-FNA), a selective µ-opioid receptor antagonist, highlighting their potential for further development.

Taking into account the satisfactory biological activity of the obtained EM analogs, we decided to check their neuroprotective profile. To explore this effect, we used a CORT-induced in vitro model of depression using SH-SY5Y cells. Additionally, we proposed an anti-inflammatory study by assessing the ability to reduce nitric oxide (NO), tumor necrosis factor alpha (TNF-α), and interleukin 6 (IL-6) production in macrophage RAW 264.7 cells damaged by lipopolysaccharide (LPS) and interferon gamma (IFN-γ).

In this study, peptides **2**, **3**, **6**, and **7** showed the best increase in cell survival in the CORT-injured cell model. However, further studies of the mechanism revealed that the greatest neuroprotective potential concerns analogs **3** and **7**. These peptides most effectively suppressed the changes caused by CORT administration. They significantly reduced the release of lactate dehydrogenase (LDH) and inhibited reactive oxygen species (ROS) production and [Ca^2+^]_i_ release. Peptides improved mitochondrial function and enhanced ATP production while reducing apoptosis and caspase-3 activity. Additionally, they modulated the balance of pro- and anti-apoptotic genes (Bax and Bcl-2, respectively) expression. We also suggested that the attenuation of the cytotoxic effect of CORT additionally resulted from the peptide’s ability to increase the mRNA and protein levels of brain-derived neurotrophic factor (BDNF). Moreover, in the LPS- and IFN-γ-induced damage model of RAW 264.7 cells, analogs **3** and **7** reduced the inflammatory response.

## 2. Materials and Methods

### 2.1. Peptide Synthesis

Peptides were synthesized by standard solid-phase procedures, as described before [18,19,20], using techniques for 9-fluorenylmethoxycarbonyl (Fmoc)-protected amino acids on MBHA Rink-Amide peptide resin (100–200 mesh, 0.80 mM/g, Novabiochem) and 2-(1H-benzotriazol-1-yl)-1,1,3,3-tetramethyluronium tetrafluoroborate (TBTU) as a coupling agent. Crude peptides were purified by preparative reversed-phase HPLC on a Vydac C18 column (10 μm, 22 × 250 mm) equipped with a Vydac guard cartridge. For purification, a linear gradient of 0–100% acetonitrile containing 0.1% TFA over 15 min. at a flow rate of 15 mL/min. was used. The purity of the final peptides was verified by analytical HPLC employing a Vydac C18 column (5 μm, 4.6 × 250 mm) and the solvent system of 0.1% TFA in water (A) and 80% acetonitrile in water containing 0.1% TFA (B). A linear gradient of 0–100% solvent B over 25 min. at a flow rate of 1 mL/min. was used for the analysis. The absorbance was monitored at 214 nm. The final purity of all peptides was >98%. Calculated values for protonated molecular ions were in agreement with those determined by FAB mass spectrometry. The physicochemical data of tested peptides are presented in Table 1. The combination of 2D 1H NMR measurements and molecular modeling calculations were published earlier [18,19,20].

### 2.2. Cell Culture and Treatment

The human neuroblastoma cell line (SH-SY5Y) was purchased from the European Collection of Cell Cultures (ECACC) and cultivated at 37 °C in a humidified atmosphere of 5% CO_2_ in the air with Dulbecco’s Modified Eagle Medium (DMEM): Nutrient Mixture F-12 (Ham’s) (1:1) (Gibco/Life Technologies, Carlsbad, CA, USA) containing 2 mM glutamine, antibiotics (100 U/mL penicillin and 100 µg/mL streptomycin) (Sigma-Aldrich, St. Louis, MO, USA), and 10% (*v*/*v*) heat-inactivated fetal bovine serum (FBS, Biological Industries, Beit-Haemek, Israel) according to the previously described protocol [17,21]. The mouse macrophage-like RAW 264.7 cells were purchased from American Type Culture Collection (ATCC, Manassas, VA, USA) and maintained in DMEM (Gibco™, Grand Island, NY, USA) supplemented with 10% bovine calf serum (BCS, Gibco™, Grand Island, NY, USA) and 1% penicillin–streptomycin (Sigma-Aldrich, St. Louis, MO, USA) [21]. The number of cells was assessed under a phase-contrast microscope based on the exclusion of trypan blue dye. The cultured cells were maintained for 3 days to allow for adhering to the plates. Subculture was performed using 0.25% trypsin/EDTSA (Gibco/Life Technologies, Carlsbad, CA, USA) after the cells reached confluence.

The tested peptides and CORT were dissolved in sterile DMSO and further diluted with a culture medium. The final concentration of DMSO in cell cultures was less than 0.1% (*v*/*v*). Controls without and with 0.1% DMSO were performed in each experiment. At the used concentration, DMSO did not affect the observed parameters.

To measure the cytotoxic effect of the tested compounds, the SH-SY5Y cells were incubated for 24 h with different doses of peptides (0.01, 0.1, 1 µM) and CORT (10, 25, 50, 100, 200, 400, 600 µM) alone.

In a cell model imitating stress-induced damages, peptides and CORT were co-incubated for 24 h. To study the inflammatory effect, 24 h after RAW 264.7 seeding, LPS/IFN-γ and peptides were added for the next 24 h.

### 2.3. Cytotoxic and Neuroprotective Activities of Peptides

The MTT (3-(4,5-dimethylthiazol-2-yl)-2,5-diphenyltetrazolium bromide) assay, which measures the activity of cellular dehydrogenases as an indicator of cell viability and cytotoxicity, was performed according to the Mosmann method [22] with minor modifications. It is a quantitative method based on the reduction in tetrazolium yellow dye named MTT by metabolically active cells to purple formazan crystals. The concentration of formazan was measured calorimetrically. Exponentially growing cells were seeded into 96-well plates at a density of approximately 2 × 10^4^ cells/well and left to grow for 24 h under controlled conditions (37 °C; 5% CO_2_). Subsequently, SH-SY5Y cells were exposed for 24 h to peptides (0.01–1 µM) and CORT (200 µM). After 24 h of incubation, cells were incubated with 20 µL of MTT tetrazolium salt solution (5 mg/mL in phosphate-buffered saline, PBS) for 1.5 h under a controlled condition (37 °C; 5% CO_2_). Following the incubation time, the medium was aspirated, and DMSO (100 µL) was added to each well to dissolve the crystals, whose absorbance was measured at 560 nm using FlexStation 3 Multi-Mode Microplate Reader (Molecular Devices, LLC, San Jose, CA, USA). The experimental design included vehicle controls (non-treated cells) and blanks (wells without cells). The cell viability rates were determined as a percentage relative to the viability of the control group (non-treated cells). The data were expressed as mean ± SEM of three to four independent experiments.

Additionally, to evaluate the release of lactate dehydrogenase (LDH) into the SH-SY5Y cell culture medium upon cell damage or lysis, we used a commercially available CytoTox 96^®^ Non-Radioactive Cytotoxicity Assay (Promega Corporation, Madison, WI, USA) according to the manufacturer’s instructions. The assay measures LDH activity based on the conversion of a tetrazolium salt into a red formazan product. The color intensity of the red formazan is directly proportional to the amount of LDH released and hence the number of damaged/lysed cells. Briefly, SH-SY5Y cells were seeded into 96-well plates at a density of approximately 2 × 10^4^ cells/well and left to grow for 24 h under controlled conditions (37 °C; 5% CO_2_). Then, they were exposed to peptides (0.01–1 µM) and CORT (200 µM) for 24 h. At the end of the incubation time, 50 µL of the sample was incubated with CytoTox 96^®^ Reagent in the dark for 30 min at room temperature. Then, 50 μL of Stop Solution was added, and finally, absorbance was read at 490 nm at 37 °C using FlexStation 3 Multi-Mode Microplate Reader (Molecular Devices, LLC., San Jose, CA, USA). The experimental design included control group (non-treated cells) and blanks (wells without cells). The release of LDH was determined as a percentage relative to the LDH leakage of the control. The data were expressed as mean ± SEM of three independent experiments.

### 2.4. Trypan Blue Exclusion Assay

We further investigated the impact of co-incubated peptides and CORT on cell viability using the Trypan blue exclusion assay to validate the results. Trypan blue is a diazo dye that selectively enters only dead cells, binding to intracellular proteins and staining them blue. Conversely, viable cells with undamaged cellular membranes resist staining and do not take up the dye, allowing them to be distinguished from the dead cells. Briefly, SH-SY5Y cells were seeded at a density of 4 × 10^5^ cells/well in 6-well plates and left to grow for 24 h under controlled conditions (37 °C; 5% CO_2_). After 24 h of incubation with various concentrations of the tested compounds, cells were detached by trypsinization, centrifuged, and the pellet was resuspended in a fresh growth medium. From the cell suspension, 15 µL was transferred to an Eppendorf tube, to which an equivalent volume of 0.4% trypan blue dye (Sigma-Aldrich, St Louis, MO, USA) was added and loaded onto the counting chambers. The experimental design included vehicle control (non-treated cells). The cell viability rates were determined as a percentage relative to the viability of the vehicle control (non-treated group). The data were expressed as mean ± SEM of three independent experiments. Cells were counted using an inverted microscope (magnification 40×, Motic Images Plus version 3.0).

### 2.5. Determination of Cell Morphology Using Wright–Giemsa Staining

The morphological changes in SH-SY5Y cells induced by CORT and peptides were analyzed by Wright–Giemsa (Merck KGaA, Darmstadt, Germany) staining according to the method described by Dlugosz-Pokorska et al. [23]. The dye stains the cytoplasm an orange to pink color, while the nucleus is blue to purple, depending on the acidity of the cytoplasmic content. Briefly, SH-SY5Y cells were seeded at a density of 4 × 10^5^ cells/well in 6-well plates and left to grow for 24 h under controlled conditions (37 °C; 5% CO_2_). After 24 h of incubation with various concentrations of the tested compounds, the cell culture medium was removed, and the cells were rinsed twice with PBS. The cells were then fixed with cold methanol for 5 min and washed with fresh PBS again. A staining procedure was performed using a Wright–Giemsa solution for 30 min, followed by a rinse with water and subsequent drying. Morphological changes in the SH-SY5Y cells were examined using an inverted microscope (magnification 40×, Motic Images Plus version 3.0) with a built-in camera (Motic Moticam 2300, 3.0M Pixel USB2.0, Merazet, Poznań, Poland and photographed.

### 2.6. Determination of Intracellular Reactive Oxygen Species (ROS)

Intracellular ROS levels were analyzed using 5(6)-carboxy-2′,7′-dichlorofluorescein diacetate (Carboxy-H2DCFDA, Sigma-Aldrich, St. Louis, MO, USA) according to the method previously described by Silva et al. [24] with minor changes. Carboxy-H2DCFDA is a cell-permeant and non-fluorescent indicator of ROS that, in the presence of ROS, undergoes oxidation, resulting in a green fluorescent signal. To assess intracellular ROS generation, SH-SY5Y cells were seeded at a density of 2 × 10^4^ cells/well in 96-well black/clear bottom plates and left to grow for 24 h under controlled conditions (37 °C; 5% CO_2_). Afterwards, they were rinsed with HEPES-buffered salt solution (HBSS, ThermoFisher Scientific, Waltham, MA, USA) to remove traces of the medium, and carboxy-H2DCFDA at a final concentration of 10 μM in culture medium without FBS and phenol red was added. After 30 min of incubation at 37 °C, the carboxy-H2DCFDA-containing medium was removed, cells were washed with HBSS, and fresh medium containing peptides and CORT was added. After incubation, fluorescence intensity was monitored at wavelengths of 485 nm (excitation) and 535 nm (emission) using a FlexStation 3 Multi-Mode Microplate Reader (Molecular Devices, LLC, San Jose, CA, USA). The experimental design included control wells and menadione (MQ, 50 µM) as a positive control. The data was analyzed and expressed as a percentage of the control (non-treated cells).

### 2.7. Determination of Mitochondrial Membrane Potential Changes (∆Ψm)

Changes in the mitochondrial membrane potential (∆Ψm) were measured using the commercially available JC-10 assay kit (Sigma-Aldrich, St. Louis, MO, USA) according to the manufacturer’s instructions. The 5,5′,6,6′-tetrachloro-1,1′,3,3′-tetraethylbenzimidazolocarbocyanine iodide (JC-10) is a cationic and lipophilic dye that can selectively penetrate mitochondria. It forms reversible red fluorescent JC-10 aggregates (λex = 540/λem = 590 nm) in the mitochondria of healthy cells with a polarized mitochondrial membrane. When ΔΨm is depolarized, the mitochondria fail to retain JC-10, leading to the dye reverting to its monomeric green fluorescent form (λex = 490/λem = 525 nm), indicating cells with inherently low ΔΨm, suggestive of apoptosis. Briefly, SH-SY5Y cells were seeded at a density of 2 × 10^4^ cells/well in 96-well black/clear bottom plates and left to grow for 24 h under controlled conditions (37 °C; 5% CO_2_). Following treatment with CORT and peptides for 24 h, SH-SY5Y cells were loaded with 50 μL/well of JC-10 for 30 min. After incubation, 50 µL/well of Assay Buffer was added. Then fluorescence intensity was monitored at wavelengths of λex = 490/λem = 525 nm (green fluorescence monomer) and λex = 540/λem = 590 nm (red fluorescence aggregates), respectively, using FlexStation 3 Multi-Mode Microplate Reader (Molecular Devices, LLC, San Jose, CA, USA). The data was analyzed and expressed as a ratio of red (λex = 540/λem = 590 nm) to green (λex = 490/λem = 525 nm) fluorescence. The measured ratio values were normalized to the control (non-treated cells) and expressed as a percentage of control. A mitochondrial uncoupler, carbonyl cyanide-4-(trifluoromethoxy)phenylhydrazone (FCCP, Sigma-Aldrich, St. Louis, MO, USA) was used as a positive control (50 µM, 30 min treatment at 37 °C).

### 2.8. Measurement of Adenosine Triphosphate (ATP) Levels

An adenosine triphosphate (ATP) is the primary energy carrier in all metabolically active cells; thus, it is widely used as a marker of cell viability. When cells undergo necrosis or apoptosis, the existing ATP is quickly degraded, and its concentration rapidly declines. Therefore, measuring ATP levels can provide a reliable indication of the number of viable cells in a sample. To investigate the changes in cellular ATP levels, a luminescence ATP Detection Assay System ATPLite assay kit (PerkinElmer, Waltham, MA, USA) was used according to the manufacturer’s instructions. Briefly, SH-SY5Y cells were seeded at a density of 2 × 10^4^ cells/well in 96-well white/clear bottom plates and left to grow for 24 h under controlled conditions (37 °C; 5% CO_2_). Following treatment with peptides for 24 h, SH-SY5Y cells were loaded and shaken with 50 μL/well of cell lysis solution for 5 min. After that, cells were treated with 50 μL/well of substrate solution and shaken for another 5 min. Then, after 10 min of incubation in the dark, the luminescence intensity was measured using a microplate reader FlexStation 3 Multi-Mode Microplate Reader (Molecular Devices, LLC., San Jose, CA, USA). The data was analyzed and expressed as a percentage of the control (non-treated cells).

### 2.9. Determination of Intracellular Calcium Ion [Ca^2+^]_i_ Level

The intracellular calcium [Ca^2+^]_i_ level, as a secondary cytotoxic hallmark, was determined using the fluorescent probe Fura-2-acetoxymethyl ester, Fura 2-AM (Fura-2 pentakis(acetoxymethyl) ester, Sigma-Aldrich, St. Louis, MO, USA) according to the described method [25,26], with minor changes. This membrane-permeable calcium indicator is used to measure cellular calcium concentrations by fluorescence. After entering the cell, cytosolic esterases convert Fura 2-AM to its active form, enabling it to bind calcium and emit fluorescence (380 nm). Briefly, SH-SY5Y cells were seeded at a density of 2 × 10^4^ cells/well in 96-well plates and left to grow for 24 h under controlled conditions (37 °C; 5% CO_2_). Following treatment with peptides and CORT for 24 h, SH-SY5Y cells were washed with HEPES buffer solution (HBSS, pH 7.4), and loaded with a loading buffer (HEPES buffer solution containing 135 mM NaCl, 5 mM KCl, 1 mM CaCl_2_, 1 mM MgCl_2_, 25 mM glucose, 10 mM HEPES, 0.1% bovine serum albumin, pH 7.4) and 0.5 µM of Fura 2-AM for 60 min in total darkness at 37 °C. Afterwards, they were rinsed with HBSS to remove traces of the medium with free Fura 2-AM. The fluorescence intensity was quantified at an excitation of 340/380 nm and an emission of 510 nm and monitored using FlexStation 3 Multi-Mode Microplate Reader (Molecular Devices, LLC, San Jose, CA, USA). The relative [Ca^2+^]_i_ level was calculated from the ratio of the emission intensities with excitations of 340 nm and 380 nm (ratio 340/380).

### 2.10. Measurement of Caspase-3 Activity

Caspase-3 activity was measured using a colorimetric assay kit (Millipore, APT165, Billerica, MA, USA) following the manufacturer’s instructions. Briefly, SH-SY5Y cells were seeded at a density of 4 × 10^5^ cells/well in 6-well plates and left to grow for 24 h under controlled conditions (37 °C; 5% CO_2_). Following treatment with peptides and CORT for 24 h, SH-SY5Y cells were harvested and lysed in an ice-cold lysis buffer, then centrifuged. Supernatants were collected, and protein concentrations were measured using the Bradford protein assay. The isolated supernatant was incubated with a reaction buffer containing the caspase-3 substrate acetyl-Asp-Glu-Val-Asp-p-nitroaniline (Ac-DEVD-pNa) at 37 °C for 2 h. Absorbance was measured at 405 nm at 37 °C using FlexStation 3 Multi-Mode Microplate Reader (Molecular Devices, LLC., San Jose, CA, USA). The experimental design included a p-nitroaniline (pNA) standard curve, control (non-treated cells), and blanks (wells without cells). The caspase activity was determined using a standard curve and expressed as the relative fold of caspase-3 activity of each sample compared with the control. The data were expressed as mean ± SEM of three independent experiments.

### 2.11. Real-Time Assessment of Cell Death Type

Cell death type was found using the RealTime-GloTM Annexin V Apoptosis and Necrosis Assay (Promega, Mannheim, Germany), according to the manufacturer’s guidelines and a previous paper [23]. The test kit included Annexin V-LgBiT (1000×), Annexin V-SmBiT (1000×), CaCl_2_ (1000×), Annexin V NanoBiT^®^ Substrate (1000×), and Necrosis Detection Reagent (1000×). Briefly, SH-SY5Y cells were seeded at a density of 2 × 10^4^ cells/well in 96-well white plates and left to grow for 24 h under controlled conditions (37 °C; 5% CO_2_). Following treatment with peptides and CORT for 24 h, 100 μL of 2× detection reagent was added to each well. Then, the plate was shaken for 30 s (at 500–700 rpm) and incubated in a humidified incubator. The luminescence signal and fluorescence (excitation of 485 nm, emission of 530 nm) intensity were analyzed using FlexStation 3 Multi-Mode Microplate Reader (Molecular Devices, LLC, San Jose, CA, USA). The measured values were compared to the control.

### 2.12. Quantitative Real-Time PCR Assay (qPCR)

The mRNA levels of Bax, Bcl-2, caspase-3, and BDNF genes were analyzed by qPCR. Briefly, SH-SY5Y cells were seeded on the 6-well plates (4.0 × 10^5^ cells/well) and then incubated with peptides and CORT. Total RNA was extracted using the Total RNA Mini Kit (A&A Biotechnology, Gdynia, Poland) according to the manufacturer’s protocol. The concentration of RNA was measured using a sensitive single-tube fluorimeter for the fluorescence-based quantitation of nucleic acids and proteins (QuantiFluor^®^ RNA System, Promega, Mannheim, Germany). TranScriba Kit (A&A Biotechnology, Gdynia, Poland) was used for cDNA synthesis. Amplification of cDNA was performed using Real-Time 2×-PCR SYBR Master Mix (A&A Biotechnology, Gdynia, Poland) in Stratagene MX3005P QPCR System (Agilent Technologies, Inc., Santa Clara, CA, USA) according to the manufacturer’s instructions. Glyceraldehyde 3-phosphate dehydrogenase (GAPDH) was used as an internal reference gene to normalize the expression of investigated genes. The expression levels of the tested genes were determined by the 2^−∆∆CT^ method [27]. The primer sequences used for qPCR are listed in Table 2.

### 2.13. Estimation of Brain-Derived Neurotrophic Factor (BDNF) Using ELISA

Various treatments can influence BDNF levels in SH-SY5Y cells. Thus, we checked the impact of peptides on CORT-injured cells’ condition. Cells were seeded at a density of 4 × 10^5^ cells/well in 6-well plates and left to grow for 24 h under controlled conditions (37 °C; 5% CO_2_). Following treatment with peptides and CORT for 24 h, the supernatants were collected and centrifuged at 150× *g* at 4 °C to remove floating cells. Then, the prepared supernatants were immediately added to a 96-well plate coated with a primary antibody specific to human BDNF according to the manufacturer’s instructions (Human BDNF SimpleStep ELISA^®^ Kit, Abcam, Cambridge, Great Britain) and our previous study [17]. Briefly, 50 μL of all samples or standard was added to the appropriate wells, with 50 μL of the Antibody Cocktail. The plate was sealed and shaken (400 rpm) for 1 h at room temperature. Then, the plate was washed with 1X Wash Buffer PT (250 μL × 3 times), and TMB substrate solution was added and shaken (400 rpm) for 10 min in the dark. The color developed in proportion to the amount of bound BDNF. Finally, the stop solution changed the color from blue to yellow, and the intensity was measured at 450 nm. A standard curve was run for each assay, and all standards or samples were run in triplicate.

### 2.14. Western Blot Analysis

Western blot analysis was used for assaying BDNF protein expression. Briefly, SH-SY5Y cells were seeded at a density of 4 × 10^5^ cells/well in 6-well plates and left to grow for 24 h under controlled conditions (37 °C; 5% CO_2_). Following treatment with peptides and CORT for 24 h, cells were washed 2 times with ice-cold PBS, and then 160 µL of ice-cold RIPA lysis buffer [with a cocktail of protease inhibitors (Hoffman-La Roche Ltd., Basel, Switzerland), pH 7.4] was added. Cells were incubated on ice for 15 min. Lysates were then centrifuged at 12,000× *g* for 10 min at 4 °C, and the supernatants were used as the total cell lysates. The protein content in the supernatants was quantified using the Bio-Rad protein assay kit. Equal amounts of protein were separated on 10% sodium dodecyl sulfate (SDS)–polyacrylamide gels and transferred onto a membrane using the Trans-Blot Turbo system (Bio-Rad, California, CA, USA), which allows for rapid and efficient protein transfer. Non-specific binding was blocked with tris-buffered saline Tween-20 (TBS-T: 150 mM NaCl, 20 mM Tris-HCl, 0.1% Tween 20, pH 7.5) containing 10% (*w*/*v*) nonfat milk for 1 h at room temperature. Then, the membrane was incubated overnight at 4 °C with one of the following specific primary antibodies: Vinculin (1:1000 dilution; Cell Signaling Technology, Danvers, MA, USA) or BDNF (1:1000 dilution; Cell Signaling Technology, Danvers, MA, USA), with 1% (*w*/*v*) bovine serum albumin. After incubation, the membranes were washed six times per 5 min with TBS-T. In the next step, membranes were incubated with HRP-conjugated secondary antibodies (1:1000 dilution; Cell Signaling Technology, Danvers, MA, USA) in TBS-T with 5% nonfat milk powder for 1 h at room temperature. Following six washes with TBS-T, the protein bands were detected with the Westar Supernova reagent from Cyanagen and visualized on a CCD camera in G:Box Chemi XR5, Syngene System. The band intensity was quantified by NIH ImageJ (version 3.0) densitometric analysis. Values of unstimulated samples were set to 1.0.

### 2.15. Secretion of Pro-Inflammatory Mediators

The level of nitric oxide (NO) production was determined in RAW 264.7 cells. Briefly, after cells’ attachment, they were treated with LPS (O55: B5 from Escherichia coli, Millipore Sigma, Darmstadt, Germany) at 1 µg/mL and 100 U/mL IFN-γ in medium with 3% of FBS for 24 h, and then the peptides were added for additional 24 h. After cells’ treatment, the medium was collected and the accumulation of NO metabolite in the cell culture supernatant was measured using Griess reagent (1% sulfanilamide and 0.1% naphthylethylenediamine dihydrochloride; Sigma-Aldrich), where 50 µL of the supernatant was mixed with 50 µL of Griess reagent (40 mg/mL) in a 96-well plate. After incubation at room temperature and darkness for 10 min, the absorbance was measured at 540 nm. Cells treated with LPS/IFN-γ without peptides were used as the positive control of the inflammatory response. Simultaneously, in the supernatants after RAW 264.7 cell treatment, the protein concentrations of interleukin 6 (IL-6) (Mouse IL6 ELISA kit, Biorbyt Ltd., Cambridge, UK) and tumor necrosis factor α (TNF-α) (Mouse TNFalpha ELISA kit, Biorbyt Ltd., Cambridge, UK) were determined by absorbance detection using ELISA kits, following the manufacturer’s instructions.

### 2.16. Statistical and Data Analyses

All data in this study were expressed as mean ± SEM of at least three to four independent experiments performed in triplicate. Statistical analyses were performed using one-way analysis of variance (ANOVA), followed by Dunnett’s multiple range test. Differences were considered statistically significant at *p* < 0.05. All the statistical analyses and constructions of graphs were performed using statistical software GraphPad Prism 6 (San Diego, CA, USA).

## 3. Results

### 3.1. Peptide Synthesis

Our study explored CORT-induced damage in SH-SY5Y cells and LPS- and IFN-γ-induced inflammation in RAW 264.7 cells, evaluating changes following the administration of peptides (Table 1), which in our previous studies showed analgesic potential [18,19,20]. The peptides were characterized by modifications at position 2, where proline was replaced with unnatural amino acids featuring six-membered heterocyclic rings, such as piperidine-2-, 3-, and 4-carboxylic acids ((*S*)-Pip, (*R*)-Nip, and Inp, respectively). Additionally, the two best analogs (peptides 4 and 8) in receptor studies were modified at position 1 by replacing tyrosine with 2,6-dimethyltyrosine (Dmt) [20].

### 3.2. Cytotoxicity and Neuroprotection

#### 3.2.1. Effects of CORT and Peptides Alone on Cell Viability

**Figure 2:**
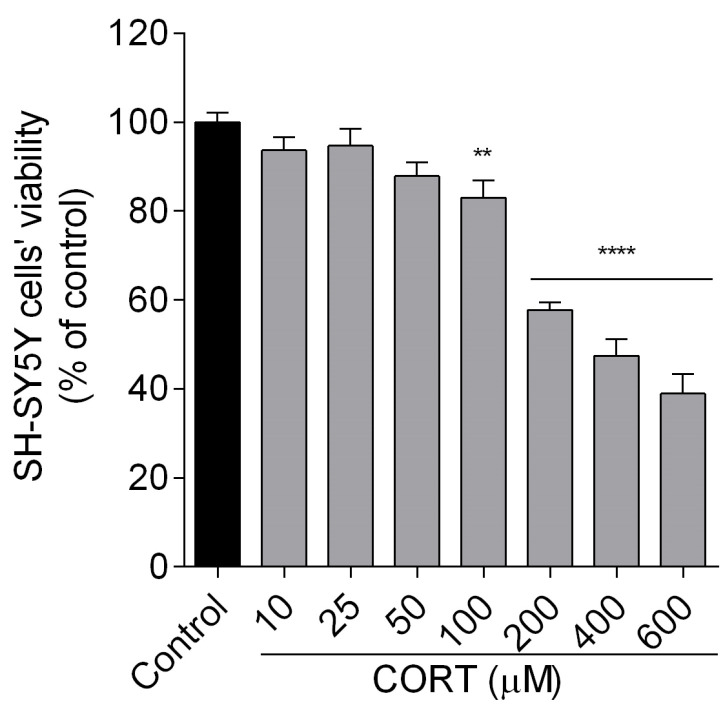
Effects of corticosterone (CORT) on the viability of SH-SY5Y cells. Neuroblastoma cells were exposed to CORT at different concentrations (10–600 µM) for 24 h. Cell viability was measured by MTT assay. All the data were presented as mean ±SEM of three independent experiments. The statistically significant changes are indicated by asterisks (ANOVA, Student’s t-test): ** *p* < 0.01, **** *p* < 0.0001 compared to control.

First, we established a CORT-induced damage model. Cultured SH-SY5Y cells were exposed to an increasing concentration of CORT (10–600 µM) for 24 h, and cell viability was evaluated using an MTT assay. As shown in Figure 2, CORT inhibited the metabolic activity of the cells in a concentration-dependent manner. The highest concentration (600 µM) reduced the cell viability to about 40%. However, at 200 µM of CORT, there was a significant reduction in cell viability by approximately 60%, and this concentration was chosen in the proposed in vitro model of cell stress to test the neuroprotective effects of peptides.

**Figure 3:**
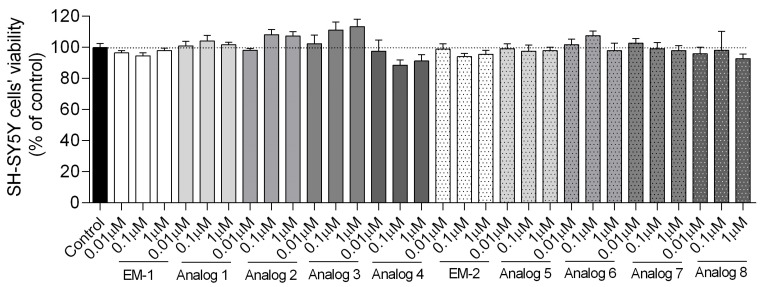
Effects of peptides on the viability of SH-SY5Y cells. Neuroblastoma cells were exposed to peptides at different concentrations (0.01–1 µM) for 24 h. Cell viability was measured by MTT assay. All the data were presented as mean ± SEM of three to four independent experiments.

To investigate the influence of peptides on cell viability, we treated SH-SY5Y cells with various concentrations of peptides (0.01–1 µM) for 24 h and examined cell viability with the MTT assay. This concentration range was chosen to evaluate both low-dose and near-maximal effects without inducing non-specific toxicity. It provided a sufficient window to observe dose-dependent responses in cell viability. None of the tested concentration range peptides showed a statistically significant toxic effect (Figure 3). Interestingly, we identified that analogs **1**, **2**, **3** (0.1 and 1 µM), and **6** (0.1 µM) induced a modest increase in cell viability. Since our aim was to identify peptides with potential protective effects, and because this range did not result in cytotoxicity, we did not extend the study to include toxic concentrations.

#### 3.2.2. Effects of Peptides on the Cell Viability of CORT-Injured SH-SY5Y Cells

**Figure 4:**
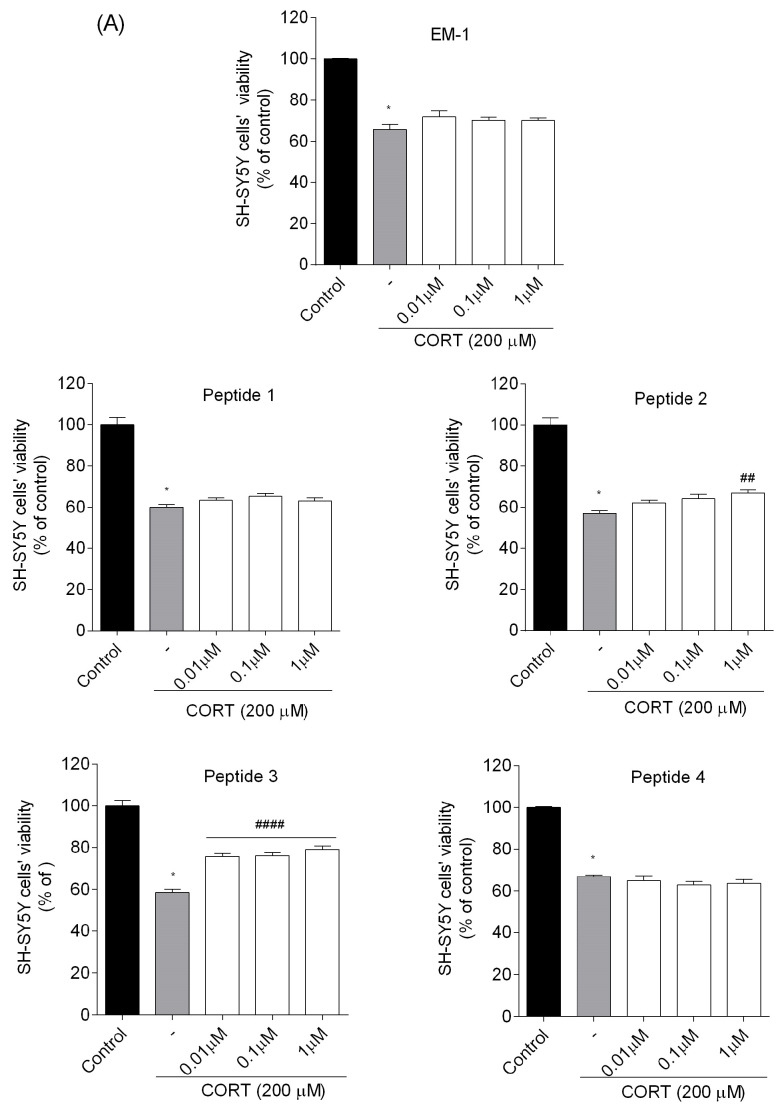

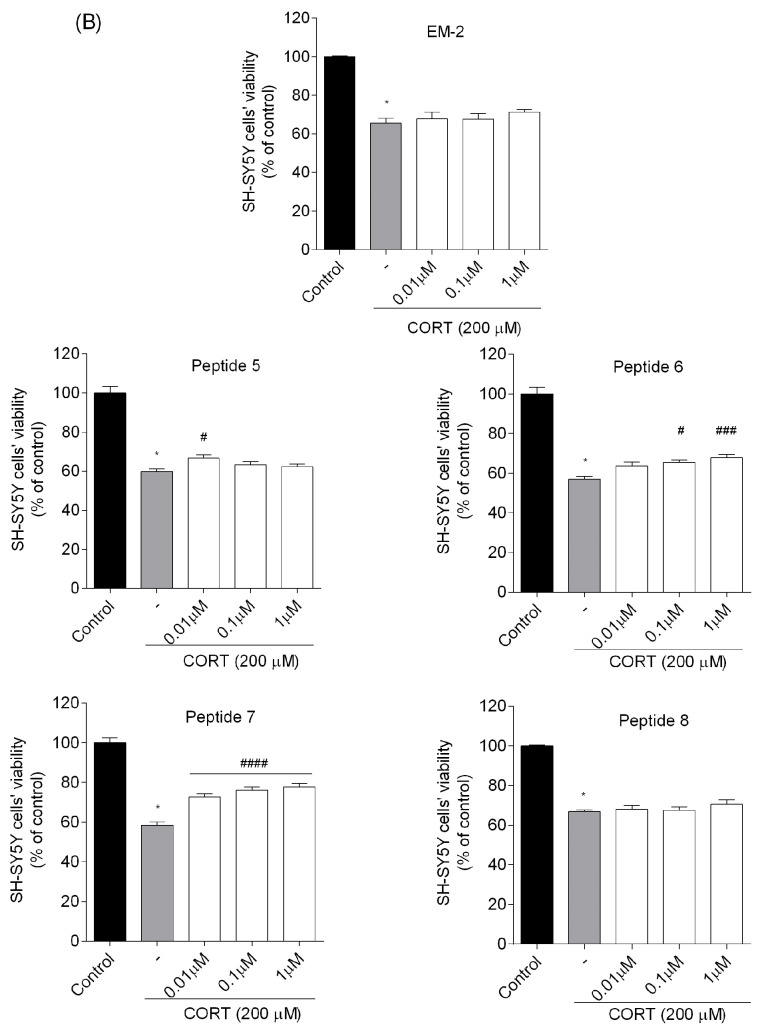
Effects of (**A**) EM-1 analogs, and (**B**) EM-2 analogs on the viability of CORT-injured SH-SY5Y cells. Neuroblastoma cells were exposed to peptides (0.01–1 µM) and CORT (200 µM) for 24 h. Cell viability was measured by MTT assay. All the data were presented as mean ± SEM of three–four independent experiments. The statistically significant changes are indicated by asterisks (ANOVA, Dunnett’s test): * *p* < 0.0001 as compared to control, and # *p* < 0.05, ## *p* < 0.01, ### *p* < 0.001, #### *p* < 0.0001 as compared to CORT-treated cells.

Since none of the peptides were cytotoxic, they were all tested to assess their neuroprotective potential in an injured model of CORT-induced neuronal damage. When SH-SY5Y cells were exposed to peptides (0.01–1 µM) in the presence of CORT (200 μM) for 24 h, the co-treated cells showed a significant increase in cell viability compared to those treated with CORT alone (Figure 4). Endomorphins, analogs **4** and **8** (incorporating Dmt in position 1 and (*R*)-Nip in position 2) did not significantly attenuate CORT-induced toxicity, while analogs **1** and **5** (incorporating (*S*)-Pip in position 2) very slightly increased cell viability. However, the best results were obtained for analogs **2** and **6** (incorporating (*R*)-Nip in position 2) as well as **3** and **7** (incorporating Inp in position 2). These four peptides were examined in subsequent steps to understand the mechanism of their neuroprotective effects.

**Figure 5:**
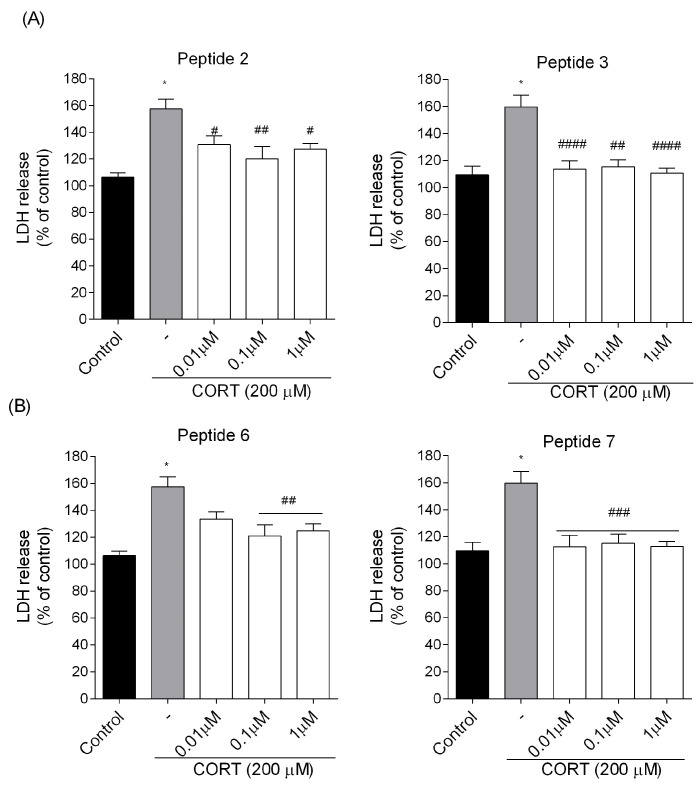
Effects of the most potent (**A**) EM-1 analogs and (**B**) EM-2 analogs on lactate dehydrogenase (LDH) release activity in SH-SY5Y cells. Neuroblastoma cells were exposed to peptides (0.01–1 µM) and CORT (200 µM) for 24 h. All the data were presented as mean ± SEM of three–four independent experiments. The statistically significant changes are donated by asterisks (ANOVA, Dunnett’s test): * *p* < 0.0001 as compared to control, and # *p* < 0.05, ## *p* < 0.01, ### *p* < 0.001, #### *p* < 0.0001 as compared to CORT-treated cells.

The MTT test results were consistent with observations regarding lactate dehydrogenase (LDH) release into the cell culture supernatant obtained from the LDH assay (Figure 5). The enzyme leakage increased by approximately 50% after exposure of cells to CORT (200 μM) when compared to the control group. Treatment with peptides (**2**, **3**, **6**, and **7**) resulted in a significant decrease in LDH release compared to the CORT injury group (Figure 5). Statistically significant changes were observed for analogs **2** and **6** (the greatest reduction in LDH release at the dose of 0.1 µM, *p* < 0.01) as well as analogs **3** (the greatest reduction in LDH release at the dose of 0.01 and 1 µM, *p* < 0.0001) and **7** (throughout the tested concentration range *p* < 0.001), compared to CORT-injured cells.

**Figure 6:**
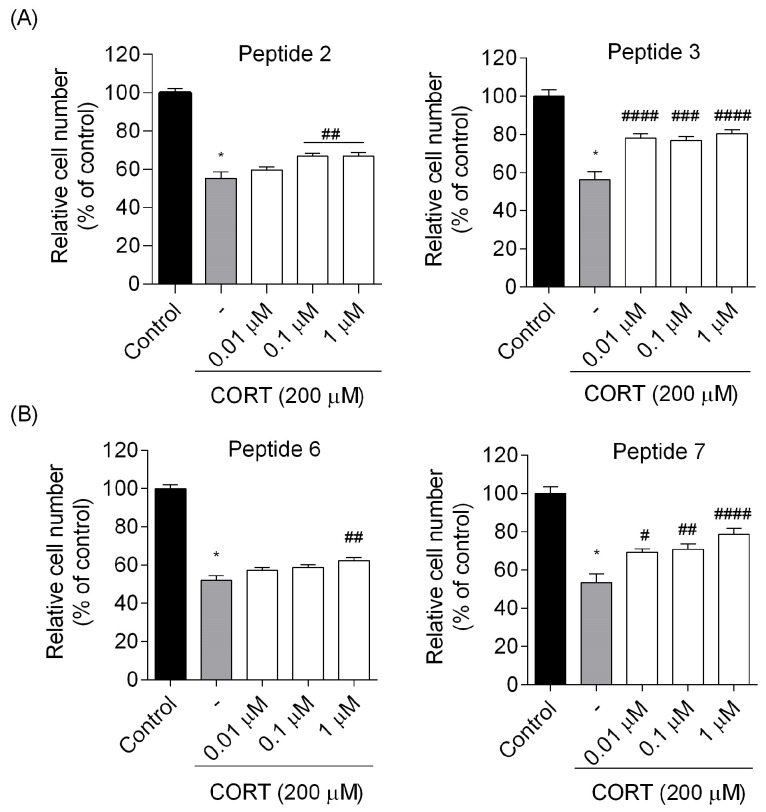
Effects of the most potent (**A**) EM-1 analogs and (**B**) EM-2 analogs on cell numbers determined in trypan blue exclusion assay. Neuroblastoma cells were exposed to peptides (0.01–1 µM) and CORT (200 µM) for 24 h. All the data were presented as mean ± SEM of three independent experiments. The statistically significant changes are indicated by asterisks (ANOVA, Dunnett’s test): * *p* < 0.0001 as compared to control, and # *p* < 0.05, ## *p* < 0.01, ### *p* < 0.001, #### *p* < 0.0001 as compared to CORT-treated cells.

To further confirm the effects of peptides on cell viability in the CORT-treated cells, the trypan blue exclusion assay was used to determine the number of viable/live cells. Again, we confirmed that treatment of cells with CORT causes a minor decrease in cell viability following 24 h incubation (Figure 6). The relative number of cells did not fall below 50% and remained in the range of 52–57% in any experimental series as compared to the control (*p* < 0.0001). In contrast, incubation with peptides **2**, **3**, **6,** and **7** increased the relative cell number (Figure 6). Peptides **3** (the number of cells maintained at around 78–80% for the dose of 0.01 and 1 µM, *p* < 0.0001, and around 76% for the dose of 0.1 µM, *p* < 0.001) and 7 (~78% for the dose of 1 µM, *p* < 0.0001) exerted their most protection potential against CORT-induced injury, ameliorating its toxic effects.

**Figure 7:**
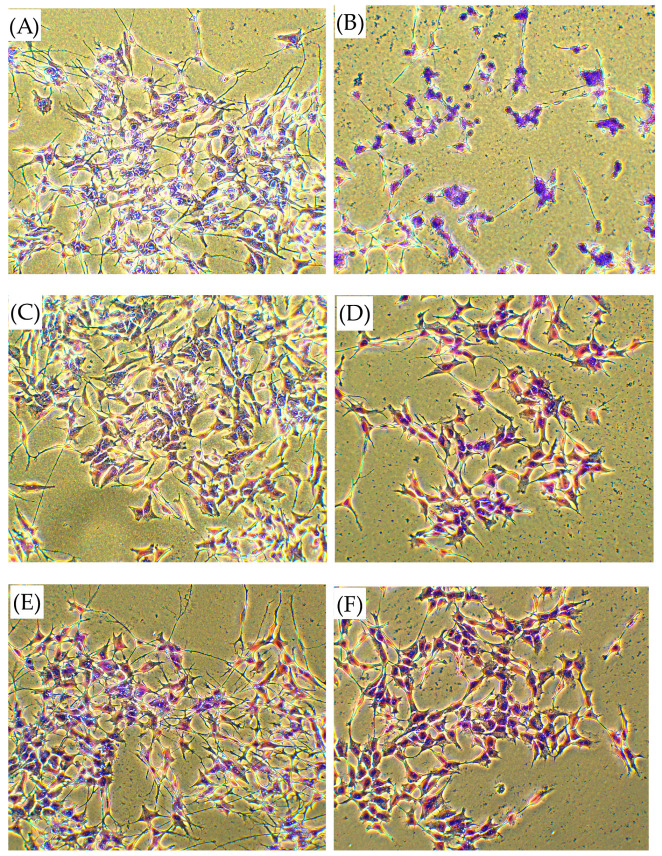
Phase-contrast images of morphological detection of apoptosis using Wright–Giemsa staining on CORT-injured SH-SY5Y cells treated with peptide 3: (**A**) control group, (**B**) CORT-injured group, (**C**) peptide **3** (1 μM), (**D**) CORT (200 μM) + peptide **3** (1 μM), (**E**) peptide **7** (1 μM), and (**F**) CORT (200 μM) + peptide **7** (1 μM). Unprocessed phase-contrast images are represented in Supplementary Figure S1. Images were obtained in phase at 40× magnification using phase-contrast microscopy.

To assess the morphological indications of injury elicited by CORT in SH-SY5Y cells and inhibited by peptides subjected to the injury model, we conducted observations of cell morphology using Wright–Giemsa staining and light microscopy. As shown in Figure 7A, the cells in the control group (non-treated) had typical morphology, characterized by non-polarized cell bodies with few truncated processes. The vesicular nucleus and prominent nucleolus were visible. The CORT-injured cells displayed evident morphological and quantitative signs of apoptosis, such as cellular shrinkage and the appearance of nuclear fragmentation, manifesting an atypical cellular morphology (Figure 7B). The cells were smaller in size, the cytoplasm was dense, and the organelles were more tightly packed. We noticed the formation of membrane vesicles, separation from the surface, and aggregation. The CORT-injured model exhibited a significantly greater population of injured cells compared to the peptide co-treatment groups. After peptide addition, the observed phenomenon of cellular shrinkage and disruption became less pronounced, reducing the morphological indications of cellular damage when compared to the CORT-injured cells. Below are the pictures for the selected peptide **3** at the dose of 1 µM administrated alone (Figure 7C) and as a co-treatment with CORT (Figure 7D). Wright–Giemsa staining indicated that peptide **3** had a protective effect on damaged SH-SY5Y cells by reducing morphological changes typical of apoptosis.

### 3.3. Effects of Peptides on Oxidative Stress Induced by CORT

#### 3.3.1. Determination of Intracellular Reactive Oxygen Species (ROS)

**Figure 8:**
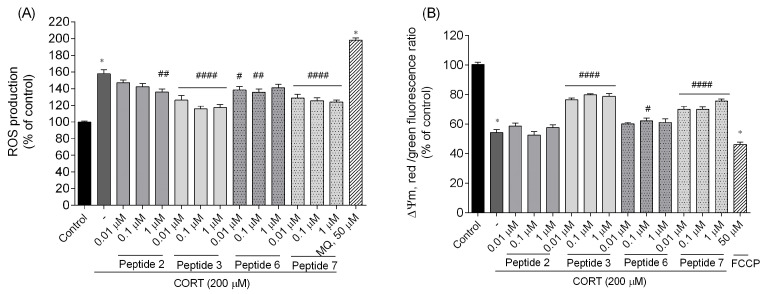
Effects of peptides on (**A**) intracellular ROS generation and (**B**) ΔΨm measurement. Neuroblastoma cells were exposed to peptides **2**, **3**, **6**, and **7** (0.01–1 µM) and CORT (200 µM) for 24 h. In the ROS experiment, menadione (MQ, 50 µM) was used as a positive control. In the ΔΨm measurement, carbonylcyanide-p-trifluoromethoxyphenylhydrazone (FCCP, 50 µM) was used as a positive control. ΔΨm was expressed as a ratio of red (λex = 540/λem = 590 nm) to green (λex = 490/λem = 525 nm) fluorescence. All the data were presented as mean ± SEM of three independent experiments. The statistically significant changes are indicated by asterisks (ANOVA, Dunnett’s test): * *p* < 0.0001 as compared to control, and # *p* < 0.05, ## *p* < 0.01, #### *p* < 0.0001 as compared to CORT-treated cells.

To determine whether the most promising peptides in this series (analogs **2**, **3**, **6**, and **7**) attenuate cell damage by blocking ROS generation, we detected the intracellular level of ROS using carboxy-H2DCFDA fluorescent dye. Treatment of SH-SY5Y cells with CORT (200 µM) for 24 h resulted in a significant increase in ROS levels compared with the control group (non-treated cells) to about 160% (Figure 8A). Menadione (MQ, 50 µM) was used as a positive control, and it also caused an elevation of ROS by approximately 100% vs. control. Treatment of CORT-injured cells with selected peptides showed a decrease in ROS levels. The reduction in ROS activities was most significant for peptides **3** and **7** (by the decreasing ability to reduce intracellular ROS levels, *p* < 0.0001). It can also be seen that in both cases, the lowest dose of 0.01 µM showed a slightly smaller tendency to eliminate the changes induced by CORT administration. Analogs **2** and **6** suppressed ROS generation noticeably weaker. For peptide **2** (at the dose of 0.01 and 0.1 µM) and peptide **6** (at the dose of 1 µM), inhibition was not statistically significant. However, these results suggest that the inhibition of the CORT-induced increase in ROS activity may be related to the neuroprotective potential of selected peptides. Peptides may exert neuroprotective effects, at least in part, through antioxidant mechanisms, potentially mitigating CORT-induced oxidative damage.

#### 3.3.2. Determination of Mitochondrial Membrane Potential Changes (∆Ψm)

The generation of ROS can significantly affect mitochondrial membrane potential (ΔΨm), causing its depolarization. These changes lead to the impairment of mitochondria through the inhibition of the electron transport chain at the complex I, resulting in ATP depletion and thus initiating the death of cells via apoptosis or necrosis as well as further damage of mitochondrial components [28,29]. To examine the protective effect of peptides **2**, **3**, **6**, and **7** (0.01–1 µM) on CORT-induced cell damage, ΔΨm, as one of the key parameters of mitochondrial function, was detected by the indicator of cell health/cytotoxicity, a JC-10 staining. The JC-10 dye concentrates in the form of red fluorescent aggregates in the mitochondrial matrix of normal/healthy cells. In contrast, in apoptotic/unhealthy cells, JC-10 diffuses out of the mitochondria, changes to the monomeric form, and stains cells green [30]. In our research, CORT (200 µM) induced a strong depolarization of ΔΨm, because we observed a significant decrease in the red/green fluorescence ratio to about 54% compared to the control group (*p* < 0.0001) (Figure 8B). Similarly, exposure of cells to carbonylcyanide-p-trifluoromethoxyphenylhydrazone (FCCP, positive control, 50 µM) significantly reduced the ratio to 46%, indicating the occurrence of ΔΨm depolarization again. Peptide treatment attenuated unfavorable changes, and the ΔΨm increased in the case of analogs **3** and **7**. Notably, peptide **6** also contributed to a modest improvement in membrane potential; however, at only a concentration of 0.1 µM, it demonstrated a significant enhancement (*p* < 0.05). Peptide **2** did not reduce excessive polarization and restore mitochondrial condition.

#### 3.3.3. Measurement of Adenosine Triphosphate (ATP) Levels

**Figure 9:**
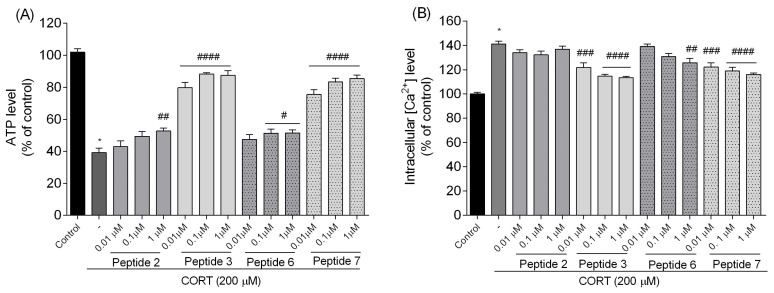
Effects of peptides on (**A**) ATP level and (**B**) the intracellular [Ca^2+^]_i_ level. Neuroblastoma cells were exposed to peptides **2**, **3**, **6**, and **7** (0.01–1 µM) and CORT (200 µM) for 24 h. All the data were presented as mean ± SEM of three independent experiments. The statistically significant changes are indicated by asterisks (ANOVA, Dunnett’s test): * *p* < 0.0001 as compared to control, and # *p* < 0.05, ## *p* < 0.01, ### *p* < 0.001, #### *p* < 0.0001 as compared to CORT-treated cells.

Adenosine triphosphate (ATP) is essential for cell survival and metabolism, and CORT-induced neurotoxicity has been linked to the impairment of ATP production [31]. Therefore, the ATP Detection Assay System ATPLite assay kit (PerkinElmer) was used to ascertain if peptides prevented ATP reduction in CORT-injured cells. We evaluated ATP changes in cells following CORT toxicity. Figure 9A shows that after CORT treatment, the ATP level significantly decreased to about 40% compared to the control. The alterations in the ATP changes were detected when peptides were administered because all of them reversed the CORT-related decrease in ATP. The data suggest that inhibition of ATP depletion may be partially involved in the neuroprotective potential of all tested peptides, with analog **3** having the most significant effects since it attenuated CORT-induced ATP depletion and remained at around 88% (*p* < 0.0001). Only the lowest dose (0.01 µM) had a less pronounced effect on eliminating CORT-induced changes. Similar alterations were observed for peptide **7**, also with a clear decrease in potential for the lowest dose. Even peptides **2** (*p* < 0.01) and **6** (*p* < 0.05) at the highest doses slightly influenced the higher ATP production.

#### 3.3.4. Determination of Intracellular Calcium Ion [Ca^2+^]_i_ Level

Intracellular calcium [Ca^2+^]_i_ overloading induced by CORT or any other type of neurotoxins disrupt normal cellular functions, leading to severe neuronal damage and cell death [2]. This process often involves the disturbance of calcium homeostasis within neurons, which is crucial for their function and survival. To examine whether peptides can change high intracellular calcium [Ca^2+^]_i_ levels in the CORT-exposed SH-SY5Y cells, the release of calcium was studied using a Fluo-2 AM fluorescence labeling assay. Figure 9B shows that CORT (200 µM) increased the intracellular [Ca^2+^]_i_ fluorescence intensity ratio by 40% compared to the control group (*p* < 0.0001). However, peptides **3**, **6** (only at the dose of 1 µM), and **7** significantly decreased the fluorescence intensities of [Ca^2+^]_i_ as compared with the CORT-injured group. The best peptides (**3** and **7**) across all tested concentrations attenuated the [Ca^2+^]_i_ overloading induced by CORT by about 20%. No significant changes were observed for peptide **2**.

### 3.4. Effect of Peptides on Apoptosis Markers Induced by CORT

#### 3.4.1. Measurement of Caspase-3 Activity

**Figure 10:**
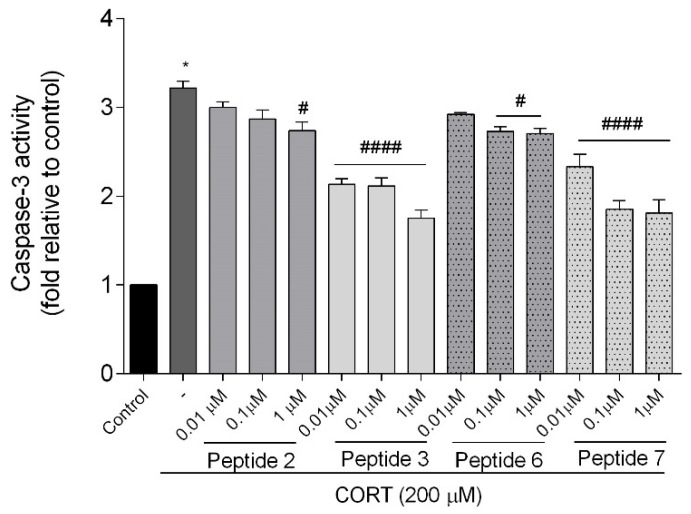
Effect of peptides on caspase-3 activity. Neuroblastoma cells were exposed to peptides **2**, **3**, **6**, and **7** (0.01–1 µM) and CORT (200 µM) for 24 h. All the data were presented as mean ± SEM of four independent experiments. The statistically significant changes are indicated by asterisks (ANOVA, Dunnett’s test): * *p* < 0.0001 as compared to control, and # *p* < 0.05, #### *p* < 0.0001 as compared to CORT-treated cells.

To check if the SH-SY5Y cell injury caused by CORT (200 µM) was mediated by apoptosis, we evaluated the activity of an important effector of apoptosis, a cysteine protease known as caspase-3 [32]. Execution and completion of apoptosis are possible after activation of caspase-3, which we confirmed in our study (Figure 10). The results showed a significant increase (3-fold) of caspase-3 activity when cells were treated with CORT (*p* < 0.0001) compared with the control. Selected peptides co-administered with CORT reduced caspase-3 activity. The attenuation of caspase-3 activity was most significant for peptides **3** and **7** (*p* < 0.0001). Peptide **6** decreased caspase-3 activity only at 0.1 and 1 µM (*p* < 0.05), while only 1 µM (*p* < 0.05) for analog **2**.

#### 3.4.2. Real-Time Assessment of Cell Death Type

**Figure 11:**
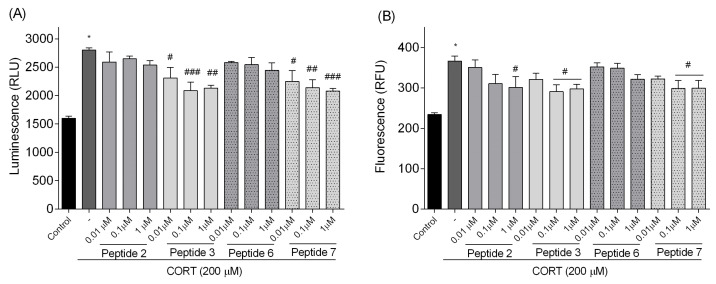
Effect of peptides on cell death by monitoring luminescence RLU (**A**) and fluorescence RFU (**B**) signals. Neuroblastoma cells were exposed to peptides **2**, **3**, **6**, and **7** (0.01–1 µM) and CORT (200 µM) for 24 h. All the data were presented as mean ± SEM of three independent experiments. The statistically significant changes are indicated by asterisks (ANOVA, Dunnett’s test): * *p* < 0.0001 as compared to control, and # *p* < 0.05, ## *p* < 0.01, ### *p* < 0.001 as compared to CORT-treated cells.

To further explore the CORT injury model, we also examine the type of SH-SY5Y cell death using RealTime-GloTM Annexin V Apoptosis and Necrosis Assay, which allows for a relative estimation of apoptosis and necrosis levels. Apoptosis (translocation of phosphatidylserine to the outer part of the cell membrane) was detected by luminescence, while necrosis (translocation of phosphatidylserine on the inner and outer part of the cell membrane) was detected by fluorescence. An increase in both luminescence and fluorescence signals indicates a loss of membrane integrity, which is characteristic of secondary necrosis following apoptosis. Figure 11A and 11B provide a relative estimation of apoptosis and necrosis levels, respectively. As we expected, CORT increased the level of RLU (luminescence signal) by about 75% and RFU (fluorescence signal) by about 56% compared to the control. Treatment with peptides rescued SH-SY5Y cells from CORT-induced changes. While the changes in peptides **2** and **6** were insignificant, peptides **3** and **7** reduced the luminescence signals (*p* < 0.05, *p* < 0.01, *p* < 0.001), confirming their protective effect and anti-apoptotic potential (Figure 11A). Significant reduction in fluorescence signal (*p* < 0.05), indicating a decrease in necrosis induced by CORT, was observed only for peptides **2** (1 µM), **3**, and **7** (0.1 and 1 µM) (Figure 11B).

#### 3.4.3. Quantitative Real-Time PCR Assay (qPCR)

**Figure 12:**
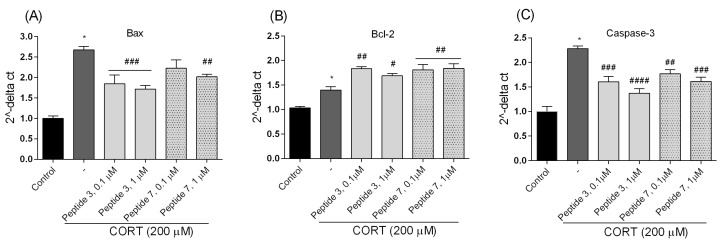
The effect of peptides **3** and **7** on Bax (**A**), Bcl-2 (**B**), and caspase-3 (**C**) mRNA expression in CORT-injured cells. Neuroblastoma cells were exposed to peptides **3** or **7** (at doses of 0.1 and 1 µM) and CORT (200 µM) for 24 h. Expression was analyzed by qPCR, relatively compared to their respective untreated controls. The expression levels of the target gene were estimated by the method after normalizing to the expression level of GAPDH. All the data were presented as mean ± SEM of three independent experiments. The statistically significant changes are indicated by asterisks (ANOVA, Dunnett’s test): * *p* < 0.0001 as compared to control, and #* p* < 0.05, ## *p* < 0.01, ### *p* < 0.001, #### *p* < 0.0001 as compared to CORT-treated cells.

Since the intrinsic apoptotic pathway is primarily controlled by proteins from the Bcl-2 and caspase families, any alterations in the expression of either pro-apoptotic or anti-apoptotic members of these families can influence the initiation of apoptosis. Moreover, in maintaining cell integrity and regulating cell viability, the balance between the expression of two genes coding for the pro-apoptotic Bax and anti-apoptotic Bcl-2 is a key element, and any disruption of this balance triggers a signaling cascade that results in cell death. The ratio of Bax to Bcl-2 serves as a regulator of intrinsic apoptotic signaling and represents a cell death switch that defines life or death orientation in response to apoptotic stimuli. Accumulation of ROS preceded mitochondrial membrane alterations and other typical apoptotic events, among which the regulation of the phosphorylation and dissemination of Bcl-2 family proteins stands out, leading to increased pro-apoptotic protein levels and reduced anti-apoptotic protein expression level [33]. In the proposed injury model, we examined the effects of peptides **3** and **7** (at doses of 0.1 and 1 µM, which were most effective) on CORT-mediated cell toxicity by measuring the expression of the Bax and the Bcl-2 using qPCR analysis. In the injury model, we observed that CORT (200 μM) significantly upregulated Bax and downregulated Bcl-2 gene expression (Figure 12A,B), confirming the promotion of apoptotic response through the mitochondrial pathway. Treatment with peptides significantly affected gene expression because CORT-mediated elevation of the Bax was suppressed. The addition of peptides reversed the CORT-mediated progression of apoptosis. A comparable situation was observed in our examination of caspase-3 gene expression levels (Figure 12C). While CORT elevated the expression of caspase-3 protein level compared to control, co-incubation with peptides significantly downregulated it. The reduction in caspase-3 mRNA expression is consistent with caspase-3 activity (Figure 10) and cell death (Figure 11) assays, confirming the anti-apoptotic properties of selected peptides.

### 3.5. Effect of Peptide on the Brain-Derived Neurotrophic Factor (BDNF) Gene and Protein Level

**Figure 13:**
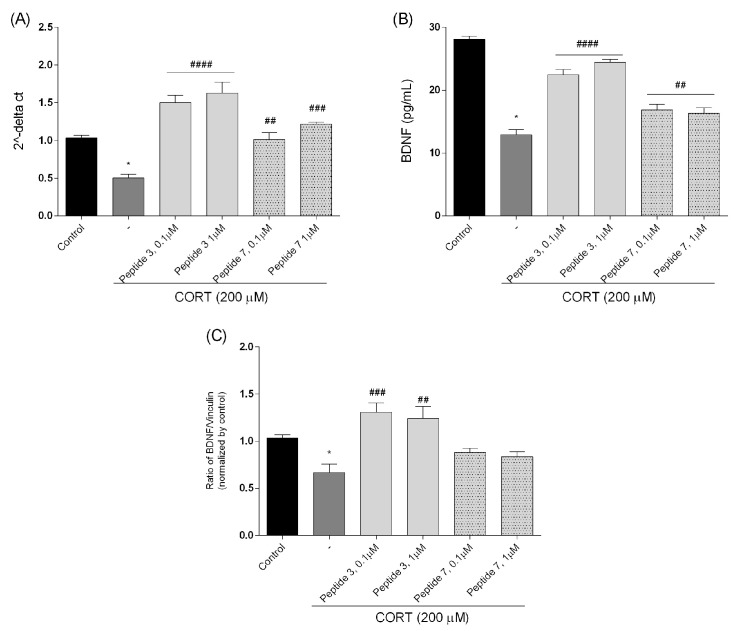
Analysis of BDNF expression at gene and protein levels, where (**A**) BDNF mRNA expression levels were measured by qPCR, (**B**) BDNF protein concentration was determined by ELISA, and (**C**) BDNF protein expression estimated by Western blot analysis. Neuroblastoma cells were exposed to peptides **3** or **7** (0.1 and 1 µM) and CORT (200 µM) for 24 h. Expression was analyzed by qPCR, relatively compared to their respective untreated controls. The expression levels of the target gene were estimated by the method after normalizing to the expression level of GAPDH. Cell lysates (20 μg protein) were analyzed for BDNF (12 kDa) activation by Western blotting using a specific BDNF antibody. The level was quantified by densitometry, and the data were normalized to values obtained from vehicle cells. All the data were presented as mean ± SEM of three independent experiments. The statistically significant changes are donated by asterisks (ANOVA, Dunnett’s test): * *p* < 0.0001 as compared to control, and ## *p* < 0.01, ### *p* < 0.001, #### *p* < 0.0001 as compared to CORT-treated cells.

Finally, in our study, we explored BDNF, a critical molecule for brain health, for the development and maintenance of the nervous system as well as neuron survival [34,35]. Level of BDNF can be changed with antidepressant treatment [36]. Higher BDNF levels after antidepressant treatment are correlated with better clinical outcomes. Patients who respond well to treatment often show significant increases in BDNF, suggesting its potential as a biomarker for treatment efficacy [37]. To characterize the effects of peptides **3** and **7** (at doses of 0.1 and 1 µM, which were most effective) on human BDNF mRNA expression, SH-SY5Y cells were treated with CORT and peptides for 24 h. Exposure of cells to CORT decreased the levels of BDNF mRNA, while treatment with the highest doses of peptides **3** and **7** significantly increased BDNF mRNA levels (*p* < 0.0001, 0.001, 0.01, respectively) (Figure 13A).

Additionally, we checked the BDNF protein level by ELISA assay. The results demonstrated that when SH-SY5Y cells were treated with CORT (200 µM, Figure 13B), cellular stress and damage were induced, leading to a decrease in BDNF protein level. The protein level in the control was about 28.11 pg/mL while the administration of CORT reduced it level to about 12.91 pg/mL. Selected peptides **3** and **7** (0.1 and 1 µM) caused an increase in the level of BDNF protein. The largest increase was observed when cells were exposed to analog **3**, while peptide **7** showed a slightly lower effect on BDNF growth, but still statistically significant (*p* < 0.01).

**Figure 14:**
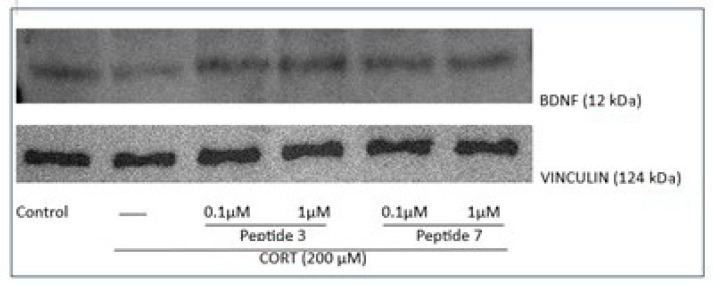
Representative Western blot bands for BDNF and loading control (vinculin) after stimulation by peptides **3** and **7** (0.1 and 1 μM) in CORT-treated SH-SY5Y cells. Cell lysates (20 μg protein) were analyzed for BDNF (12 kDa) activation by Western blotting using a specific BDNF antibody. The level was quantified by densitometry, and the data were normalized to values obtained from vehicle cells.

Western blot analysis was performed to evaluate BDNF protein expression, with vinculin used as a loading control to ensure equal protein loading across all samples. Vinculin, a cytoskeletal protein with stable expression, serves as a reliable reference for normalization in neuronal cell lysates. The analysis revealed that treatment of CORT-injured cells with peptide **3** (0.1 and 1 µM) can significantly increase the production of BDNF (BDNF, 12 kDa) (Figure 13C), which is crucial for neurotrophic effects [38]. We also observed a slight increase in the case of peptide **7**; however, this change was not statistically significant. Densitometric quantification confirmed a marked upregulation of BDNF protein levels in treated groups compared to untreated controls, supporting the neuroprotective potential of the compounds. Representative Western blot bands for BDNF and loading control are shown in Figure 14 (the original immunoblots are represented in Supplementary Figure S2).

### 3.6. Secretion of Pro-Inflammatory Mediators

A large body of evidence suggests that in the pathophysiology of depressive disorders, the pro-inflammatory mediators play important roles, and their regulation is one of the mechanisms of antidepressant treatment [39]. Research conducted on patients suffering from depression disorders indicate in serum the consistently increased levels of pro-inflammatory cytokines such as TNF-α and IL-6, with their combined activity leading to systemic inflammation. TNF-α cytokine is essential for regulating neuronal function, including synaptic activity, and via TNF-α receptors mediate neuroprotective mechanisms against neurotoxic stimuli. However, studies in mice demonstrated that TNF-α secreted from hippocampal microglia induces working memory deficits by acute stress [40], whereas increased IL-6 was found in microglia from mice that had undergone repeated social defeat stress [41], as well as in patients with treatment-resistant depression [42]. Therefore, socio-environmental cues regulate inflammation through the activation of neuroinflammatory signal transduction, with inflammatory cytokines as receptor agonists, and intracellular regulation of transcriptional response. TNF-α and IL-6 mediators have pleiotropic effects since they can be produced by several cell types, including endothelial cells, astrocytes, microglia, and neurons, but under in vitro conditions, one of the most studied inflammatory models is based on RAW 264.7 macrophages stimulated by LPS and IFN-γ [43]. Being able to secrete different types of cytokines, these cells are also producers of nitric oxide (NO), which is a messenger molecule regulating nervous and immune systems. Several studies have shown that the inhibition of NO production can be identified as one of the effects matched with antidepressant and antistress activity of some therapeutic agents [44].

**Figure 15:**
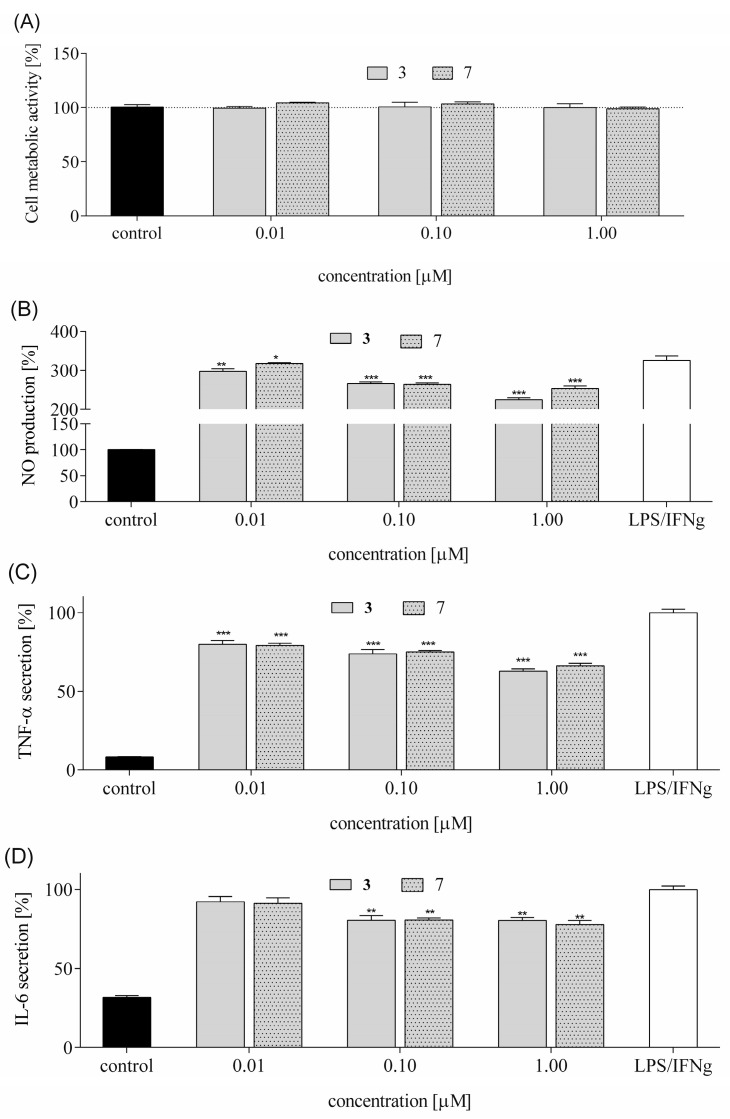
(**A**) The effect of analogs **3** and **7** on RAW 264.7 cells’ metabolic activity as determined with MTT assay after incubation with peptides for 24 h. (**B**) The effect of peptides on nitric oxide (NO) production determined with Griess reagent, (**C**) as well as the secretion of tumor necrosis factor α and (**D**) interleukin 6 as determined with ELISA. Control cells were only exposed to the vehicle. The values in each column represent the mean ± SEM, *n* ≥ 4. The statistically significant changes are indicated by asterisks (ANOVA, Dunnett’s test): * *p* ≤ 0.05, ** *p* ≤ 0.01, *** *p* ≤ 0.001 as compared to LPS/INF-γ-treated cells.

Among the studied peptides, **3** and **7** revealed the highest neuroprotective potential. First, their effect on metabolic activity was determined. As presented in Figure 15A, both compounds at concentrations 0.01–1 µM had no statistically significant effect on the metabolic activity of cells. Next, the compounds’ modulatory effect on the secretion of pro-inflammatory mediators in RAW 264.7 was studied.

Stimulation of RAW 264.7 cells significantly increased NO production—nearly fourfold compared to unstimulated controls (Figure 15B). Both peptide analogs reduced NO levels by 10–20%, with peptide **3** showing greater efficacy. LPS/IFN-γ treatment also elevated TNF-α secretion over tenfold in comparison to untreated macrophages. Both peptides similarly decreased the release of TNF-α in stimulated cells, and their inhibitory effect was concentration-dependent—0.01 µM decreased the cytokine level by 20%, whereas 1 µM decreased it by 30–35% (Figure 15C). Tested peptides also attenuated the secretion of IL-6, but here the reduction yield was much lower than that observed for TNF-α: the highest concentration reduced IL-6 by 20%, whereas for 0.01 µM, no effect was observed (Figure 15D). Overall, analogs **3** and **7** revealed anti-inflammatory potential. Since the observed anti-inflammatory potential relies on detection of released proteins, it can be suspected that the studied peptides may regulate the expression level and activity of NO synthase by affecting nuclear factor κB (NF-κB) transcriptional activity, which exerts both positive and negative influences depending on the cell type and nature of damage [45]. The protective role of NF-κB in neurons includes anti-apoptotic effects mediated by the induction of caspase inhibitors or expression of antioxidant genes. On the other hand, NF-κB activation in microglia and astrocytes leads to the production of pro-inflammatory factors like IL-6 or TNF-α, triggering neuroinflammation.

## 4. Discussion

The mechanisms underlying stress-induced damage to neurons were not fully understood, and ongoing research aimed to elucidate these mechanisms. The precise molecular and cellular pathways involved in stress-induced impairment can be complex and multifaceted. Some of the proposed mechanisms include glucocorticoid imbalance, oxidative stress, inflammation, altered neurotransmitter levels, or changes in synaptic plasticity. Here, we would like to focus on cellular changes, resulting in increased production of CORT, which have detrimental effects on neurons, including impaired stress response, mood regulation, neuroinflammation, and changes in synaptic plasticity, crucial for learning and memory. At the cellular level, stress can generate ROS and oxidative stress in neurons, leading to cellular damage involving DNA and protein damage, which may contribute to neurodegenerative conditions. As a result of stress, an inflammatory response is also generated in the brain, characterized by the activation of microglia and the release of pro-inflammatory cytokines. It is important to note that ongoing research continues to uncover new insights into the mechanisms of stress-induced neuronal damage. Using various models and techniques, including cell cultures, researchers investigate the effects of stress on neurons and their underlying mechanisms. Ultimately, a better understanding of these mechanisms helps to develop the potential treatments for stress-related neurological and psychiatric disorders. The human neuroblastoma SH-SY5Y cell line is widely used as a model cell system for studying neuronal cell death induced by various cellular stressors, including oxidative damage and mitochondrial dysfunction [16,46].

In this paper, we explored a CORT-induced in vitro injury model of stress using SH-SY5Y cells to mimic the cellular damage typically observed in depression. Although the model itself is well characterized [31,47], our study introduces a novel approach by examining peptides as possible neuroprotectants. The peptides may serve as promising candidates for mitigating neuronal alterations [16,48,49]; that is why we focused on previously obtained peptides with opioid activity, which are analogs of EMs and exhibit enhanced biological activity compared to their parent compounds [18,19,20]. The modifications introduced into the original sequence involved substituting Pro at position 2 with unnatural amino acids with six-membered heterocyclic rings, such as (*S*)-Pip, (*R*)-Nip, and Inp (Figure 1) [18,19,20]. Four peptides (analogs **2**, **3**, **6,** and **7**) were found to reduce SH-SY5Y cell stress caused by CORT in the tested concentration range, resulting in a markedly increased cell viability. Selected peptide analogs demonstrated superior neuroprotective activity compared to the parent endomorphins, which did not exhibit any protective effects in the selected neuronal damage model. This suggests that structural modifications introduced in the analogs were critical for enhancing biological efficacy, particularly in mitigating CORT-induced oxidative stress and cellular injury. Based on further research defining the mechanism of neuroprotection, we selected two peptides **3** and **7**, with the following sequence Tyr-Inp-Trp-Phe-NH_2_ and Tyr-Inp-Phe-Phe-NH_2_, respectively. The remaining peptides, especially **2** and **6**, also showed reparative effects, but the changes were less pronounced and not statistically significant; therefore, we focused primarily on peptides **3** and **7**. Those peptides reduced unfavorable morphological changes signaling apoptosis, such as cellular shrinkage, nuclear fragmentation, membrane blebbing, and separation from the surface. Peptide addition ensured healthy morphology and an unreduced number of viable cells. The CORT-induced changes, which led to the generation of ROS, mitochondrial dysfunction, ATP reduction, and disruption of calcium homeostasis, were alleviated by the administration of selected peptides. Peptides **3** and **7** showed the strongest neuroprotective effects, significantly reducing ROS levels and improving ΔΨm. Peptide **3**, in particular, effectively prevented ATP depletion. Both peptides also reduced CORT-induced intracellular Ca^2+^ overload by about 20% across all tested concentrations. Additionally, these peptides showed the highest effects on apoptotic markers induced by CORT. Co-administration of peptides with CORT significantly reduced caspase-3 activity, especially with peptides **3** and **7**, confirming their anti-apoptotic effects. These peptides also lowered luminescence and fluorescence signals, indicating reduced apoptosis and necrosis. We checked the mRNA expression levels of the pro-apoptotic Bax, anti-apoptotic Bcl-2, and caspase-3. Peptide treatment suppressed CORT-induced upregulation of Bax and caspase-3. This downregulation, consistent with reduced caspase-3 activity and cell death, confirms the anti-apoptotic effects of the selected peptides, **3** and **7**.

From our point of view, the effect of peptides on CORT-induced BDNF level suppression was also important. Brain-derived neurotrophic factor (BDNF) plays an essential role in growth, differentiation, maintenance, and cell survival. In the context of depression, previous pharmacotherapies have focused on the theory of monoaminergic dysregulation [50]. Recently, another hypothesis involving a deficiency in the neurotrophic pathway has attracted attention. This theory suggests that low levels of various neurotrophins, particularly BDNF, are linked to depressive disorders [51]. Interestingly, several factors, such as environmental, physical activity, and many natural and synthetic compounds, may modulate BDNF expression or function [51]. To date, various natural and synthetic compounds have demonstrated neuroprotective effects by boosting the expression of BDNF [52]. Rodent and human studies showed that different drugs (e.g., the selective serotonin reuptake inhibitor fluoxetine (FLX), the selective norepinephrine reuptake inhibitor reboxetine (RBX), and the tricyclic antidepressant desipramine (DMI) predominantly inhibiting norepinephrine reuptake) may selectively influence the changes in BDNF gene expression [53]. Moreover, BDNF has neuroprotective properties [34,35], which may be of great importance in the context of neurodegenerative diseases or neural injuries, because many factors may stimulate BDNF expression [47,54]. Our previous research has shown that 6-hydroxydopamine (6-OHDA)-reduced BDNF levels can be reversed by naturally occurring peptide, rubiscolin-6 (R-6) administration [21].

This time, our research showed that exposure of cells to CORT decreased the mRNA levels of BDNF, while treatment with the peptides **3** and **7** (at the dose of 0.1 and 1 µM) significantly increased BDNF mRNA levels. ELISA assay revealed that peptides increased the level of BDNF protein, and a more pronounced increase in BDNF protein levels was noted for peptide 3. Similarly, Western blot analysis showed that peptide **3** significantly increased BDNF levels, while peptide **7** caused a slight, non-significant increase.

Chronic inflammation in the central nervous system contributes to neurotoxicity by promoting the release of pro-inflammatory cytokines, which can damage neurons and disrupt neural function [55]. Therefore, we measure the levels of inflammatory cytokines such as NO, TNF-α, and IL-6 in LPS-stimulated RAW 264.7 cells. These cytokines serve as key biomarkers of neuroinflammatory activity. Elevated levels of these cytokines are associated with the breakdown of the blood–brain barrier (BBB), activation of microglia and astrocytes, and progression of neurodegenerative diseases. We observed that both peptides reduced NO levels, with peptide **3** showing greater efficacy. They reduced TNF-α secretion in a concentration-dependent manner. IL-6 secretion was also attenuated, albeit less effectively, with only the highest peptide concentration reducing levels by ~20%. These results suggest that tested peptides **3** and **7** possess anti-inflammatory properties, potentially through modulation of NO synthase expression and NF-κB activity. While NF-κB can promote neuroinflammation via cytokine release in glial cells, it may also exert neuroprotective effects in neurons by inducing anti-apoptotic and antioxidant pathways.

These findings suggest that the tested peptides exhibit promising neuroprotective properties, supporting the rationale for continued exploration of peptide-based compounds as potential therapeutic candidates.

A growing body of evidence supports the neuroprotective potential of peptides, as summarized in the reviews by Perlikowska [16] or Patel K. and Mani A. [48], which highlights various opioid peptides that modulate key pathological pathways in neurodegenerative conditions. Among opioid peptides evaluated for neuroprotection are both endogenous ligands, such as endomorphins (EM-1 and EM-2), and synthetic analogs including [D-Ala^2^, N-MePhe^4^, Gly-ol]-enkephalin (DAMGO), [D-Pen^2^, D-Pen^5^]-enkephalin (DPDPE), [D-Ala^2^, D-Leu^5^]-enkephalin (DADLE), [D-Ser^2^, Leu^5^, Thr^6^]-enkephalin (DSLET), and biphalin ((Tyr-D-Ala-Gly-Phe-NH)_2_) (see more in [16]). Notably, EM-1 and EM-2 have demonstrated antioxidant and anti-apoptotic properties by scavenging free radicals, inhibiting lipid peroxidation, and protecting mitochondrial function. They also reduced amyloid β-induced neurotoxicity in vitro and in vivo, highlighting their potential as neuroprotective agents.

Our findings underscore the critical role of position 2 modifications in enhancing the biological activity of peptide analogs. Specifically, peptides **3** and **7**, both modified with heterocyclic amino acids at this position, exhibited the most significant protective effects and demonstrated notable anti-inflammatory properties. Similar modifications in R-6 analogs also yielded promising results, suggesting a conserved mechanism of action. The incorporation of 4-carboxylic acid-containing amino acids (Inp) at position 2 appears to be a key determinant of bioactivity. These residues may enhance receptor binding affinity or selectivity, potentially through interactions with opioid receptors or other signaling pathways. The presence of a carboxylic acid moiety could contribute to improved peptide–receptor interactions via hydrogen bonding or electrostatic interactions, thereby modulating downstream biological responses. This highlights position 2 as a strategic site for rational design in the development of neuroprotective and anti-inflammatory peptide therapeutics.

The outcome of our study is the identification of peptide structures exhibiting both neuroprotective and anti-inflammatory properties. However, the most significant limitation of our findings is that the tested peptides are not able to pass the BBB, which poses a significant challenge for their direct application in treating central nervous system (CNS) disorders. Despite this limitation, we believe that further investigations are necessary to determine whether these peptides can exert neuroprotective effects in vivo, either through peripheral mechanisms or via modified delivery strategies. These efforts aim to establish a more comprehensive understanding of how opioid peptides may contribute to neuroprotection and stress resilience.

## 5. Conclusions

Our study focused on peptides structurally related to EMs, modified at position 2 with heterocyclic amino acids. Among the tested analogs, peptides **3** (Tyr-Inp-Trp-Phe-NH_2_) and **7** (Tyr-Inp-Phe-Phe-NH_2_) showed the most pronounced protective effects against CORT-induced cytotoxicity. These peptides improved cell viability, preserved healthy morphology, and significantly reduced ROS levels, mitochondrial dysfunction, intracellular Ca^2+^ overload, and ATP depletion. Both peptides also demonstrated anti-apoptotic activity by reducing caspase-3 activity and modulating the expression of Bax, Bcl-2, and caspase-3 genes. Additionally, they restored BDNF mRNA and protein levels, with peptide 3 showing a stronger effect, suggesting a role in neurotrophic support. Their anti-inflammatory potential was confirmed by reduced NO, TNF-α, and IL-6 levels, likely mediated through modulation of NO synthase and NF-κB signaling pathways.

Our findings highlight the significance of position 2 modifications in peptide analogs, particularly those incorporating heterocyclic amino acids. Interestingly, similar modifications in R-6 analogs also showed promising activity [21], suggesting that the amino acid at position 2 may play a critical role in modulating biological function. This position could influence receptor binding affinity, potentially involving opioid receptors, and warrants further investigation to elucidate its mechanistic contribution to the observed bioactivity.

Overall, our findings support the therapeutic potential of peptides **3** and **7** as neuroprotective agents. These results align with recent reviews highlighting the promise of peptide-based strategies in neurodegenerative and stress-related disorders.

## Figures and Tables

**Table 1:** Physicochemical data of endomorphin analogs [18,19,20].

No.	Sequence	HPLC ^a^	FABS-MS		
		(t_r_)	Formula	MW	[M+H]+
	Tyr-Pro-Trp-Phe-NH_2_ (EM-1)	17.30	C_34_H_37_N_6_O_5_	610	611
**1**	Tyr-(*S*)-Pip-Trp-Phe-NH_2_	17.52	C_35_H_39_N_6_O_5_	624	625
**2**	Tyr-(*R*)-Nip-Trp-Phe-NH_2_	16.32	C_35_H_39_N_6_O_5_	624	625
**3**	Tyr-Inp-Trp-Phe-NH_2_	16.82	C_35_H_39_N_6_O_5_	624	625
**4**	Dmt-(*R*)-Nip-Trp-Phe-NH_2_	18.13	C_37_H_43_N_6_O_5_	652	653
	Tyr-Pro-Phe-Phe-NH_2_ (EM-2)	16.21	C_32_H_36_N_5_O_5_	571	572
**5**	Tyr-(*S*)-Pip-Phe-Phe-NH_2_	17.18	C_33_H_38_N_5_O_5_	585	586
**6**	Tyr-(*R*)-Nip-Phe-Phe-NH_2_	16.41	C_33_H_38_N_5_O_5_	585	586
**7**	Tyr-Inp-Phe-Phe-NH_2_	16.47	C_33_H_38_N_5_O_5_	585	586
**8**	Dmt-(*R*)-Nip-Phe-Phe-NH_2_	17.55	C_35_H_42_N_5_O_5_	613	614

^a^ HPLC elution on a Vydac C18 column (5 μm, 4.6 × 250 mm) using the solvent system of 0.1% TFA in water (A)/80% acetonitrile in water containing 0.1% TFA (B) and a linear gradient of 0–100% solvent B over 25 min at a flow rate of 1 mL/min. Data from Refs. [18,19,20].

**Table 2:** Primer sequences for qPCR.

Gene	Primer Sequence	
	Forward	Reverse
Bax	ACCCGGTGCCTCAGGATGCGT	GGCAAAGTAGAAAAGGGCGAC
Bcl-2	CATGCTGGGGCCGTACAG	GAACCGGCACCTGCACAC
BDNF	TGCAGGGGCATAGACAAAAGG	CTTATGAATCGCCAGCCAATTCTC
Caspase-3	GAAGCTACCTCAAACTTCC	CAGCATCACTGTAACTTGCT
GAPDH	GTCGCTGTTGAAGTCAGAGGAG	CGTGTCAGTGGTGGACCTGAC

## Data Availability

The original contributions presented in this study are included in the article/Supplementary Material. Further inquiries can be directed to the corresponding author.

## Supplementary Materials

The following supporting information can be downloaded at: https://www.mdpi.com/article/10.3390/biomedicines13071774/s1. Figure S1. Unprocessed version of Figure 7A–F. Figure S2. Brain-derived neurotrophic factor (BDNF) stimulation by peptides 3 and 7 (at the dose of 0.1 and 1 μM) in CORT-treated SH-SY5Y cell. Figure S3. Three additional immunoblots.
