# Supplementary material for: Reduction in SH-SY5Y Cell Stress Induced by Corticosterone and Attenuation of the Inflammatory Response in RAW 264.7 Cells Using Endomorphin Analogs
Source: Biomedicines. 2025 Jul 20;13(7):1774. doi: 10.3390/biomedicines13071774 (PMC12292617; doi:10.3390/biomedicines13071774)
Supplement: Supplementary file 1 [file biomedicines-13-01774-s001.zip › biomedicines-3714436-supplementary.pdf]

biomedicines

Supplementary Materials

Reduction in SH-SY5Y cell stress induced by corticosterone and attenuation of the inflammatory response in RAW 264.7 cells using endomorphin analogs

Renata Perlikowska [1]*, Angelika Długosz-Pokorska [1], Małgorzata Domowicz [2], Sylwia Grabowicz [1], Mariusz Stasiołek [2], Małgorzata Zakłos-Szyda[3]

[1] Department of Biomolecular Chemistry, Faculty of Medicine, Medical University of Lodz, Mazowiecka 6/8, 92-215 Lodz, Poland; renata.perlikowska@umed.lodz.pl (R.P.); angelika.dlugosz@umed.lodz.pl (A.D.-P.), sylwiagrabowicz.farm@gmail.com *(S.G.),
[2] Department of Neurology, Faculty of Medicine, Medical University of Lodz, Kosciuszki Street 4, 90-419 Lodz, Poland; malgorzata.domowicz@umed.lodz.pl (M.D.), mariusz.stasiolek@umed.lodz.pl (M.S.)
[3] Institute of Molecular and Industrial Biotechnology, Faculty of Biotechnology and Food Sciences, Lodz University of Technology, Stefanowskiego 2/22, 90-537 Lodz, Poland; malgorzata.zaklos-szyda@p.lodz.pl (M.Z-S.)
* Correspondence: renata.perlikowska@umed.lodz.pl

Contents:

- Unprocessed version of Figure 7A-F. Morphological changes in the SH-SY5Y cells were examined using an inverted microscope (magnification 40×, Motic Images Plus version 3.0) with a built-in camera (Motic Moticam 2300, 3.0M Pixel USB2.0) and photographed. ……………………………………………………………………..pages 2-3

- The original immunoblots…………………………………………………page 3

 https://doi.org/10.3390/xxxxx

Corresponding to Fig. 7A-F

A) B)

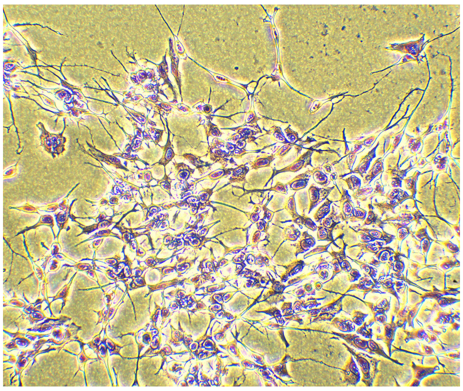

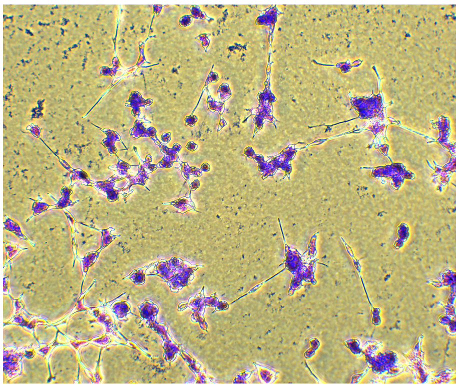

C) D)

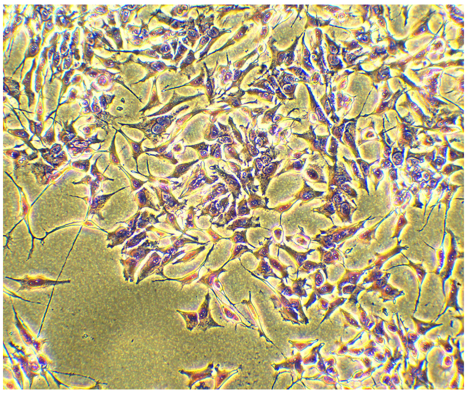

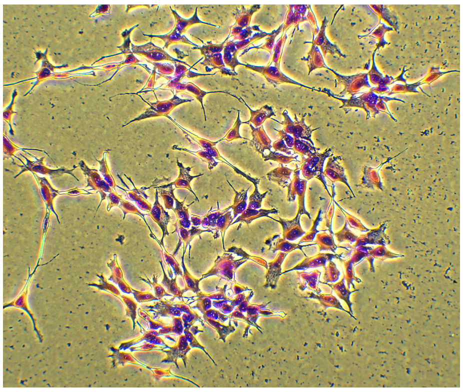

E) F)

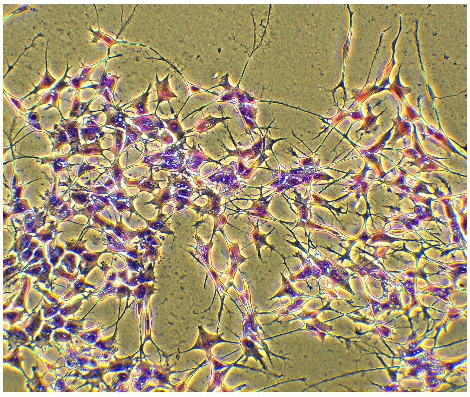

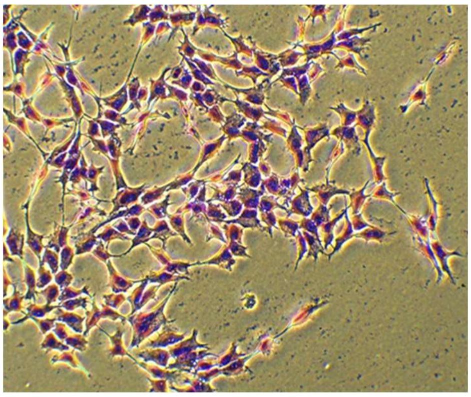

Figure S1. Phase contrast images of morphological detection of apoptosis using Wright- Giemsa staining on CORT-injured SH-SY5Y cells treated with peptide 3: (A) control group, (B) CORT-injured group, (C) peptide 3 (1 μM), (D) CORT (200 μM) + peptide 3 (1 μM), (E) peptide 7 (1 μM), (F) CORT (200 μM) + peptide 7 (1 μM). Images were obtained in phase at 40× magnification using phase-contrast microscopy.

The original immunoblots. Protein levels were normalized to Vinculin (cropped bands are shown in red frame) based on densitometry analysis, and relative protein levels are shown.

Corresponding to Fig. 14

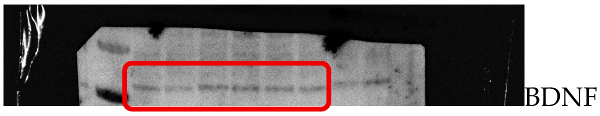
BDNF

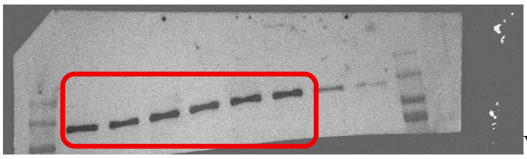
Vinculin

Figure S2. Brain-derived neurotrophic factor (BDNF) stimulation by peptides **3** and **7** (at the dose of 0.1 and 1 μM) in CORT-treated SH-SY5Y cells. Cell lysates (20 μg protein) were analyzed for BDNF activation by Western blotting using a specific BDNF antibody.

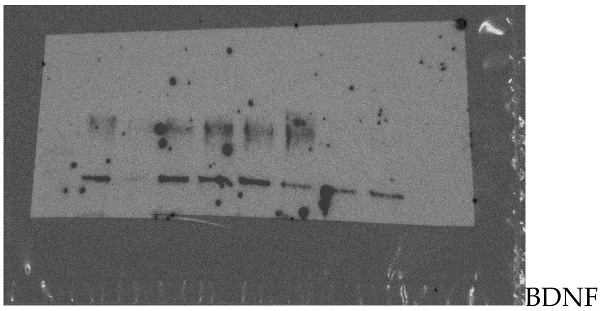
BDNF

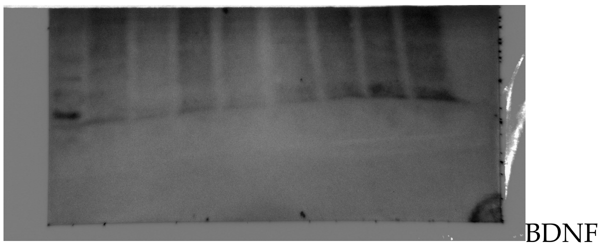
BDNF

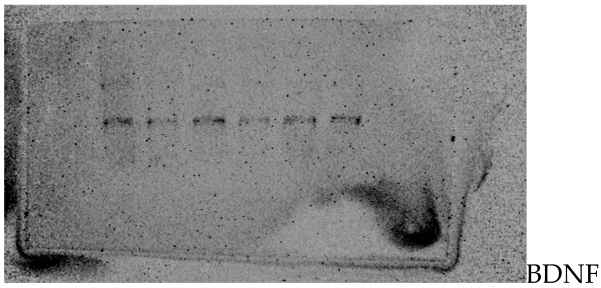
BDNF

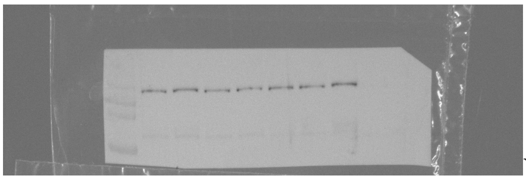
Vinculin

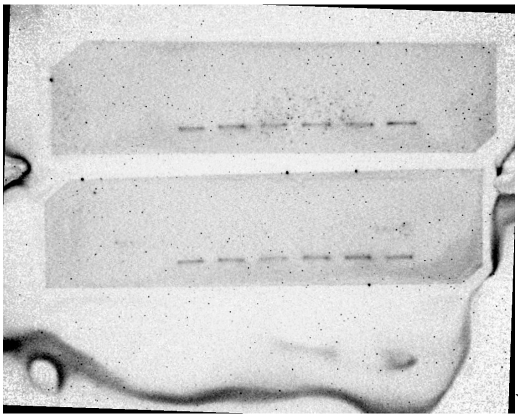
Vinculin

Figure S3. Three additional immunoblots were used for analyses of brain-derived neurotrophic factor (BDNF) stimulation by peptides **3** and **7** (at the dose of 0.1 and 1 μM) in CORT-treated SH-SY5Y cells. Cell lysates (20 μg protein) were analyzed for BDNF activation by Western blotting using a specific BDNF antibody.
